# Supplementary material for: Superlubric polycrystalline graphene interfaces
Source: Nat Commun. 2021 Sep 28;12:5694. doi: 10.1038/s41467-021-25750-w (PMC8479137; doi:10.1038/s41467-021-25750-w)
Supplement: Supplementary file 1 — Supplementary Information [file 41467_2021_25750_MOESM1_ESM.pdf]

Supplementary Information for

Superlubric Polycrystalline Graphene Interfaces

Xiang Gao, et al.

Supplementary Methods

Construction of Polycrystalline Graphene Model

The structure of the periodic polycrystalline graphene (PolyGr) surface was created using the Voronoi tessellation method developed by Shekhawat[1,2]. Compared to the grain boundaries (GBs) generated by seaming two misaligned graphene grains with annealing, this method provides physically realistic and low energy graphene GBs with dislocation content and structural properties in excellent agreement with experiment[1], as well as perfect periodicity in the lateral dimensions. Our model system comprises of two grains, one spanning the central region of the simulation cell (Marked as Grain 2 in Supplementary Fig. 1a) and the other (Marked as Grain 1 in Supplementary Fig. 1a) covers the remaining area located on its left and right sides and is connected via periodic boundary conditions in the direction perpendicular to the GB axis (marked as the x-axis in Supplementary Fig. 1a). Periodic boundary conditions are also applied for both grains along the GB axis (marked as the y-axis in Supplementary Fig. 1a). Grain 1 is positioned such that its zigzag edge resides along the GB axis and its armchair edge along x axis (equivalent to an unrolled (0,56) carbon nanotube of length $\sim$11.93 nm). This yields a grain orientation angle (the angle between the x axis and the armchair direction of the grain) of $\theta_1 = 0°$ (see Supplementary Fig. 1a). Grain 2, in turn, is constructed from an unrolled (9,51) nanotube of length $\sim$11.93 nm, whose chiral vector is oriented along the y axis, corresponding to an orientation angle of $\theta_2 = 8°$. The relaxed lengths of the two grains along the GB (y-)axis (corresponding to the unrolled nanotubes circumferences) are $l_{y,1} = 56\sqrt{3}a_{\mathrm{cc}}$ and $l_{y,2} = \sqrt{9^2 + 51^2 + 9 \cdot 51} \times \sqrt{3}a_{\mathrm{cc}} = 56.044625 \times \sqrt{3}a_{\mathrm{cc}}$ for Grain 1 and Grain 2, respectively, where a_{cc} = 1.42039 Å is the equilibrium carbon-carbon bond length in the second-generation reactive empirical bond order (REBO) potential[3]. The dimension of the PolyGr layer along the GB axis, L_y, is thus set to their average, $L_y = \left(l_{y,1} + l_{y,2}\right)/2 = 13.7825\ \mathrm{nm}$. The chosen chiral vectors introduce negligibly small strain along the GB axis, whose magnitude is $\left|l_{y,1} - l_{y,2}\right|/\left|l_{y,1} + l_{y,2}\right| \sim 4\times10^{-4}$. The corresponding strain along the x direction is discussed in detail in the last subsection of the Supplementary Methods.

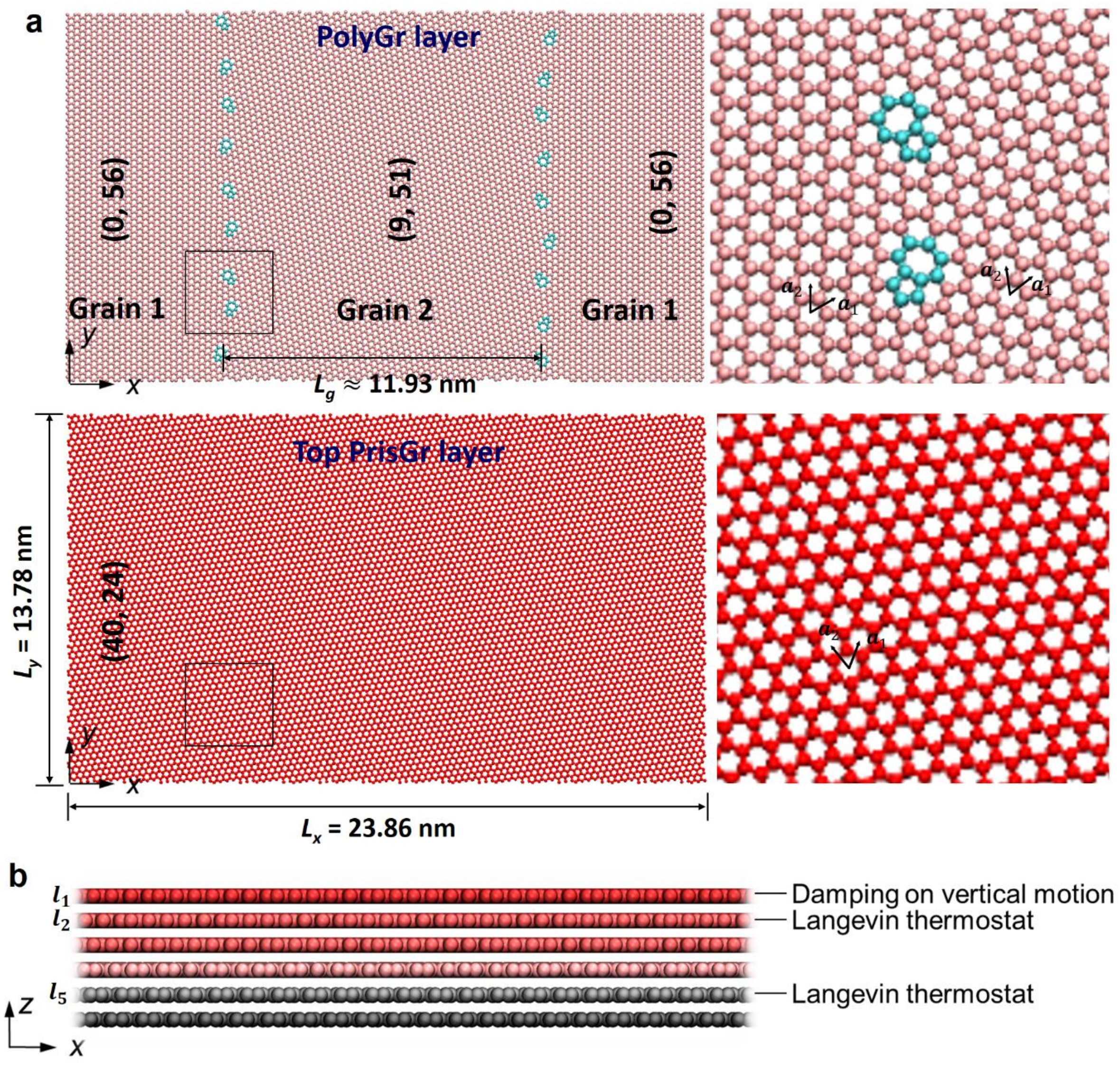

Supplementary Fig. 1. Model and simulation protocol description. (**a**) Atomic structures of the PolyGr layer (upper panel) and the topmost PrisGr layer (lower panel). Zoom-in views of the areas notated by the black squares in the left panels are provided to the right with a clear annotation of the lattice vectors representing the unrolled nanotubes that construct the various grains. (**b**) Cross sectional side view of the model system. The layers, to which damping and Langevin thermostats are applied, are explicitly denoted. The damping on the vertical motions of the atoms in the topmost layer is only applied during sliding simulations, namely not during the relaxation process. At zero temperature, the Langevin thermostats reduce to the velocity damping terms given in Eq. (S1). Pink and cyan spheres represent the hexagon and pentagon-heptagon carbon atoms in the PolyGr layer, respectively. Red and grey spheres indicate the carbon atoms in the top three PrisGr layers and the bottom two PrisGr layers, respectively. The dark red and dark grey layers are rigid.

Model System Description

To model the sliding interface formed between pristine graphene (PrisGr) and PolyGr, we constructed a model system consisting of three PrisGr layers sliding atop of a graphitic substrate consisting of a PolyGr layer supported by two PrisGr layers (see Supplementary Fig. 1b and Fig. 1 of the main text). The entire supercell is subjected to periodic boundary conditions in the lateral dimensions with minor strain effects. Similar to Grain 1 of the PolyGr layer, the two lower PrisGr substrate layers are constructed from an unrolled 23.8626 nm long carbon nanotube of chiral vector $(0, 56)$ oriented along the y-axis, yielding strains of 4×10^{-4} and 0 in the y- and x- directions, respectively. With this construction, Grain 1 of the PolyGr substrate layer is commensurate with the underlying PrisGr substrate layers, forming stable ABA stacking. The top three ABA stacked PrisGr layers are formed from unrolled ~ 23.86 nm long $(40,24)$ carbon nanotubes, whose chiral vectors are oriented along the y-axis yielding an orientation angle of $\theta_0 = 38.2°$ (lower panel of Supplementary Fig. 1a). The resulting PrisGr/PolyGr frictional interface, of surface area $\sim 23.86 \times 13.78\ \mathrm{nm}^2$, is incommensurate for both grains (misfit angles of $60° - 38.2° = 21.8°$ and $60° - (38.2° - 8°) = 29.8°$ with Grains 1 and 2, respectively, where we account for the 60° rotational symmetry of the hexagonal lattice), allowing us to isolate the dissipative effect of the GB while maintaining superlubric motion within the grain surfaces.

Such a six-layered system represents a minimal model to study dissipative effects in sheared polycrystalline interfaces. The middle layers represent the frictional interface, their adjacent layers take the role of dissipating excess energy while avoiding explicit damping of the interface of interest, and the outermost layers mimic the supporting rigid substrate and slider bulk regions. In Note 5 below we demonstrate that this minimal model produces converged results with respect to the number of layers.

The intralayer and interlayer interactions are modeled with the REBO potential[3] and the registry-dependent interlayer potential (ILP)[4-8], respectively. The latter is parameterized against density functional theory (DFT) reference calculations using the HSE06 screened-exchange functional approximation[9] including many-body dispersion (MBD) corrections[10], which provide a reliable description of van der Waals interactions in layered materials in both equilibrium and sub-equilibrium regimes[8,11]. This approach yields GBs corrugations, topographies, and energies comparable to available experimental results and DFT calculations[12-15].

Simulation Protocol

To mimic a rigid moving stage and a fixed support, in all our simulations the topmost layer was kept rigid and the atoms in the bottommost layer were fixed at their initial positions. Prior to the friction simulations, the system was subjected to structural relaxation in the absence of normal load. Notably, the geometrically generated PolyGr structures contain atomic centers with more than 3 nearest neighbors (within the distance criterion of 2.0 Å used in the ILP). Since the number of nearest neighbors in the ILP cannot exceed 3 the ILP could not be used directly. Therefore, we added a preliminary relaxation step using the Lennard-Jones (LJ) interlayer potential ($\varepsilon_{\mathrm{CC}} = 2.84$ meV and $\sigma_{\mathrm{CC}} = 3.4$ Å). The resulting structure, was then further relaxed using the ILP. In both the LJ and ILP relaxation stages the system was first minimized using the FIRE algorithm[16,17] with a force criterion of 10^{-3} eV/Å, followed by an annealing procedure, where Langevin thermostats were applied to the second and the fifth layers from the stack top (see Supplementary Fig. 1b) with a damping coefficient of 1.0 ps^{-1}. The annealing procedure consisted of: (i) raising the temperature of the system linearly from 10 K to 1000 K during 50 ps; (ii) maintaining the temperature at 1000 K for 100 ps (for the ILP calculation) or 200 ps (for the LJ calculation); (iii) cooling the system down linearly to 0 K in 50 ps; followed by (iv) a 50 ps simulation period at 0 K. The annealed configurations exhibited maximal forces of the order of 10^{-9} eV/Å, much lower than the force criterion used during FIRE minimization, therefore another round of minimization following annealing was not required. During the relaxation process, the lateral position of the topmost rigid layer was kept fixed, whereas its vertical position was free to move. After relaxation, the in-plane strain in the PolyGr layer was partially relieved by out-of-plane deformations of the dislocations in the GBs, which protrude upward or downward.

Thereafter, the starting configurations for the sliding simulations at zero temperature under different normal loads were generated by further relaxing the system with the FIRE algorithm[16,17] using a force criterion of 10^{-3} eV/Å. The normal load was applied by adding a uniform force in the range of $0 - 0.06$ nN in the vertical direction, to each atom in the topmost layer, corresponding to normal pressures of up to ~2.3 GPa. This procedure mimics a realistic scenario where a uniform load spreads from the bulk region of a sufficiently thick slider to the shear interface. As shown in Supplementary Note 5 below, the same frictional behavior is obtained for three model system thicknesses, indicating that the buffer layers residing between the loaded upper surface and the

sheared interface is sufficiently thick to provide converged results. We note that we deliberately refrain from employing a Nosé–Hoover type barostat, which couples to the dimensions of the simulation box, during our simulations for the following reasons: (i) In the vertical (out-of-plane) direction, our simulation box includes a large vacuum region to avoid spurious interactions between image stacks. The Nosé–Hoover barostat is not well suited to regulate the pressure in such a configuration mimicking an open system. Instead, we regulate the pressure by applying the external normal force to the top stack layer; (ii) In the lateral directions the system size is chosen such that the introduced strain is below 1‰. Due to the extremely high in-plane stiffness of graphene, variations of the box lateral dimensions under load can be readily neglected. The starting configurations for sliding simulations at finite temperatures were prepared by equilibrating their corresponding relaxed configurations at zero temperature with Langevin thermostats, to the target temperature for 200 ps.

During the sliding simulations, the top layer was moved with a constant velocity of v_0 = 5 m/s in the x direction (see Supplementary Fig. 1b). To remove the heat accumulated during sliding, at zero temperature, velocity damping (equivalent to Langevin thermostat at zero temperature) with a damping coefficient of 1.0 ps^{-1} was applied to the relative velocities of each atom in the second layer (slider, see Supplementary Fig. 1b) with respect to the topmost layer, and the velocities of each atom in the fifth layer (substrate, see Supplementary Fig. 1b) to mimic the energy dissipation channels through both the slider and the substrate in experiments, without affecting the dynamics of the layers at the shear plane. In addition, to avoid vibration accumulation in the vertical direction, velocity damping with the same damping coefficient of 1.0 ps^{-1} was also applied to the vertical velocities of the atoms in the topmost layer[18]. These damping terms are written as,

$$\mathbf{f}^{l_1}_{\mathrm{damp},i}(t) = -m_{\mathrm{c}}\eta v^{l_1}_z(t)\hat{\mathbf{z}},$$

$$\mathbf{f}^{l_2}_{\mathrm{damp},i}(t) = -m_{\mathrm{c}}\sum_{j=x,y,z}\eta\left(v^{l_2}_{i,j}(t) - v^{l_1}_j(t)\right)\hat{\mathbf{j}}, \quad \text{(S1)}$$

$$\mathbf{f}^{l_5}_{\mathrm{damp},i}(t) = -m_{\mathrm{c}}\sum_{j=x,y,z}\eta v^{l_5}_{i,j}(t)\,\hat{\mathbf{j}},$$

where the superscript $l_{1,2,5}$ represent the first layer, the second layer, and the fifth layer from the top to bottom, i.e. the three damped layers, m_{c} is the atomic mass of carbon, i is the ith atom in the layer, η is the damping coefficient, $v_{i,j}(t)$ is the jth Cartesian velocity component of the ith atom at time t, $\hat{\mathbf{j}} = \hat{\mathbf{x}}, \hat{\mathbf{y}}, \hat{\mathbf{z}}$ are the unit vectors in x, y and z directions, respectively. Note that damping the

relative velocities of atoms in the 2nd layer with respect to the corresponding atomic velocities of the rigid topmost layer is equivalent to damping them with respect to the 2nd layer's center-of-mass motion.

For finite temperature simulations, the Langevin thermostat, with same damping settings used in the zero temperature simulations, was employed to the 2nd and 5th layers, and damping of the vertical motion of the top rigid layer is maintained. It should be noted that during the simulations, the random forces due to the Langevin thermostat on different atoms are independent, thus the total random force on the each thermostated layer is not exactly zero at each time step. While over infinite simulation time this will be averaged out, at finite time this may introduce a non-zero center-of-mass force on the thermostated layers. Since the overall friction is very small, such a non-physical temporary force may jeopardize the physical relevance of the simulation results. Therefore, we apply the standard procedure implemented in LAMMPS[19], where at each time step the vectorial sum of all atomic random forces within a given thermostated layer (divided by the number of atoms) is subtracted from the force acting on each atom in this layer. This thermostat scheme maintains the target temperatures well under different normal loads and sliding velocities, as shown in Supplementary Fig. 2.

All simulations were performed using the LAMMPS package[19]. Typically, the sliding simulations last for 2-3 ns for zero temperature and 6-10 ns for finite temperatures to provide sufficient long steady state lateral force traces for the calculation of friction. The calculation of lateral forces and the convergence of friction are discussed in detail in Supplementary Note 1 below.

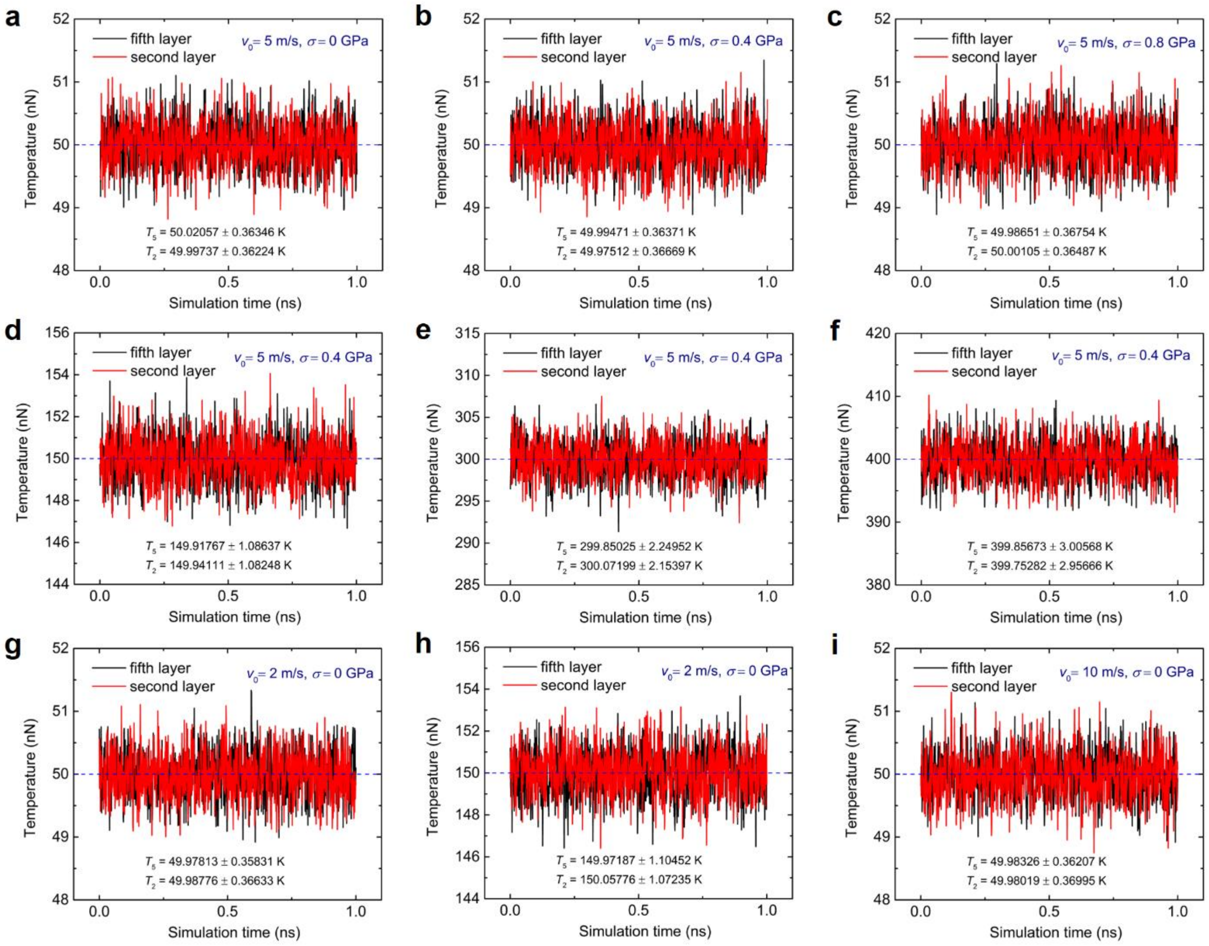

Supplementary Fig. 2. Representative instantaneous temperature profiles in the two thermostated layers under different normal loads, target temperatures, and sliding velocties. Temerature profiles for (**a**)-(**c**) utilize a target temperure of 50 K under different normal loads [(**a**) 0 GPa, (**b**) 0.4 GPa, and (**c**) 0.8 GPa] with a sliding velocity of v_0=5 m/s; (**d**)-(**f**) different target temperatures [(**d**) 150 K, (**e**) 300K, and (f) 400K) under a normal load of 0.4 GPa and a sliding velocity of 5 m/s sliding velocity, (g)-(i) different sliding velocities ((**g**), (**h**) 2 m/s and (**i**) 10 m/s) and a target temperature of (**g**), (**i**) 50K and (**h**) 150K under zero normal load. T_2 and T_5 represent the temperatures in the second layer and the fifth layer from top, where the Langevin thermostates are applied. The blue dashed lines denoting the target temperatures are shown as a guide to the eye.

Intrinsic Strain Effect in the Model System

The Voronoi tessellation method and periodic boundary condition may introduce intrinsic strain to the generated PolyGr, especially in the direction perpendicular to the GBs. To examine this intrinsic strain effect, we built a control system, where the annealed periodic PolyGr layer is cut open in the x direction by removing two $0.85\ \mathrm{nm}$ wide ribbons on both sides, as shown in Supplementary Fig. 3a. With this, the PolyGr layer in the control system does not experience periodic boundary conditions in the x-direction and is free to deform. By comparing the horizontal dimensions of the control system prior and following further annealing we can estimate the strain introduced by enforcing periodic boundary conditions. To this end, we selected two $0.43\ \mathrm{nm}$ wide ribbons in the control system (see red stripes in Supplementary Fig. 3a) and compared the distance between their centers of mass in their original configuration (right after cutting the periodic PolyGr layer) and after further annealing. For zero normal load, the calculated difference is ~0.2 Å, which is below 1‰ (part per thousand) of the horizontal dimension of the simulation box $(23.86\ \mathrm{nm})$. Moreover, we find that the average out-of-plane corrugation in the periodic PolyGr system is lower than that of the control open PolyGr system by merely 0.02 Å (see Supplementary Fig. 3b). Therefore, we conclude that the strain introduced in the PolyGr layer due to the periodic boundary condition applied in the horizontal direction is negligible and should not affect the general frictional behaviors reported in the main text.

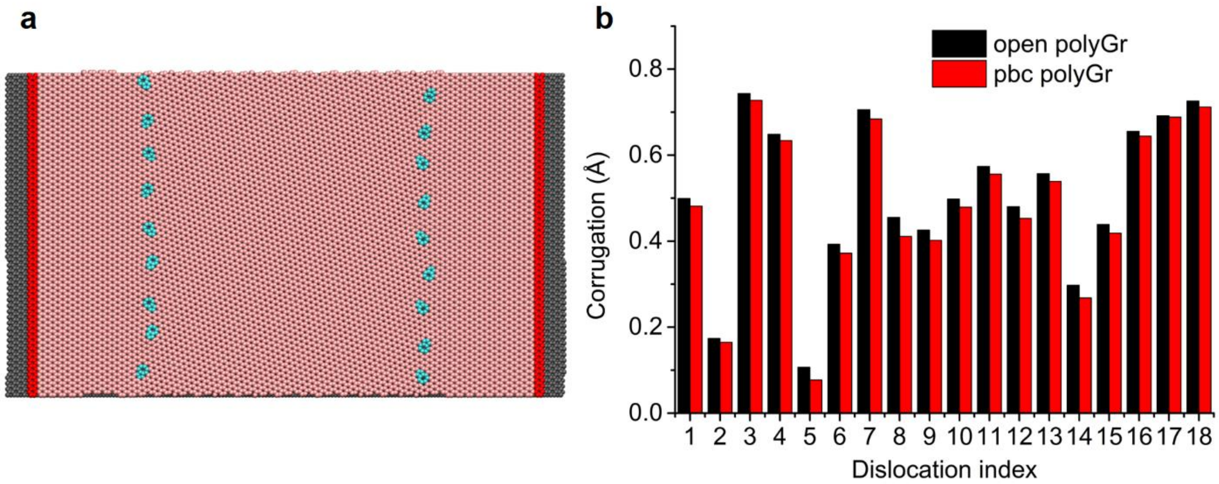

Supplementary Fig. 3. Examination of intrinsic strain effect with open PolyGr control model system. (**a**) top view of the annealed configuration of the entire six-layer stack (three top layers are not shown) with open boundary conditions applied to the PolyGr layer in the horizontal direction. The pentagon-heptagon pairs are presented by cyan spheres and the hexagonal carbon atoms in the PolyGr layer are represented by pink spheres. The two red ribbons at the edges of the PolyGr layer are used to estimate the strain induced by applying periodic boundary conditions. The underlying PrisGr layers atoms are denoted by grey spheres. (**b**) Comparison of the corrugation of each dislocation in the annealed open PolyGr system (black bars) and periodic PolyGr system (red bars) for all GB dislocations shown in panel a. The corrugation of each dislocation is chosen as the maximal out-of-plane atomic displacement (measured with respect to the average height of the two grains) in each dislocation region.

Supplementary Note 1. Lateral Force Traces and Friction Force Calculation

Lateral Force Traces

The friction can be estimated by recording the lateral forces acting in the *x* direction (sliding direction) on the top rigid sliding layer. Supplementary Fig. 4a presents the force traces for the system at zero temperature under normal loads of 0, 0.6, and 1.9 GPa, where the middle load corresponds to the peak friction and the other two represent low friction scenarios. GB dislocation buckling events are manifested as high frequency force variations over-imposed on the lower frequency oscillations of the force traces obtained under normal loads of 0 and 0.6 GPa (see inset in the middle panel of Supplementary Fig. 4a). At higher normal loads, e.g. 1.9 GPa, the buckling of dislocations is greatly suppressed, thus the force traces show no high frequency variations. With increasing temperature the lateral force traces present enhanced thermal fluctuations, manifested as pronounced high-frequency variations (see Supplementary Fig. 4b, c and d).

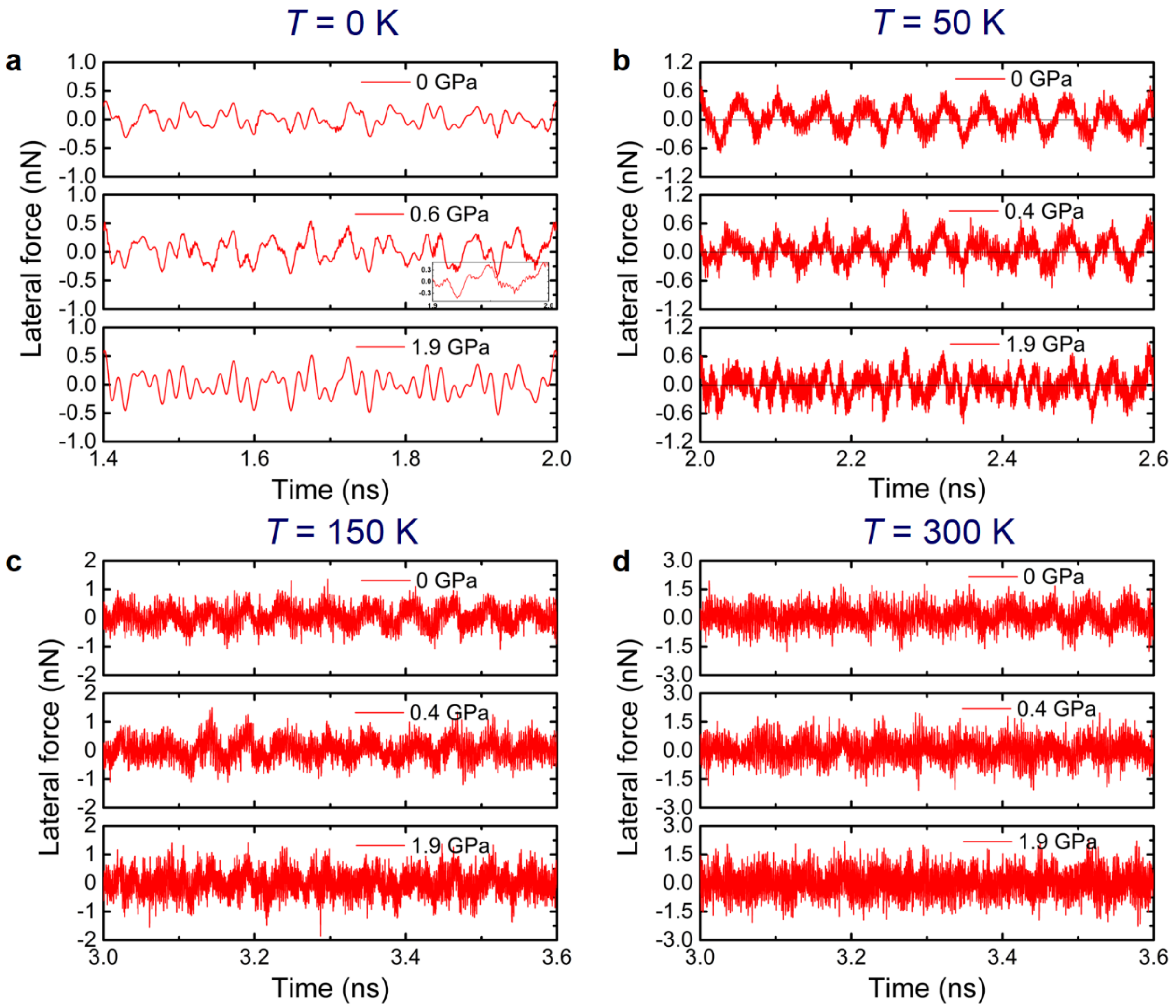

Supplementary Fig. 4. Representative lateral force traces at steady state obtained at different temperatures and normal loads. (**a**) T = 0 K under normal loads of 0, 0.6, and 1.9 GPa; (**b**) T = 50 K; (**c**) T = 150 K; and (**d**) T = 300 K under normal loads of 0, 0.4, and 1.9 GPa. Here, the simulation period of 0.6 ns corresponds to one sliding period of the PrisGr slider in the x direction. The data points are recorded with temporal resolution of 100 fs.

Friction Force Calculation and Friction Coefficient Estimation

The friction forces in Fig. 2a, b of the main text are evaluated by time-averaging the force traces discussed in the above section. To set the averaging time-window we recall that, by construction, the x-periodicity of the PrisGr slider layer, obtained by unrolling a $(40,24)$ carbon nanotube, is $7l_{\text{armchair}}$, where $l_{\text{armchair}} = 3a_{\text{cc}}$ is the periodicity of the graphene lattice in the armchair direction and $a_{\text{cc}} = 1.42039$ Å is the equilibrium C-C bond length of graphene in REBO potential. Considering the sliding velocity of $5\ \text{m/s}$, the time periodicity of the sliding is $t_0 \approx 596.6$ ps, which serves as our time-window for averaging the force traces.

In Supplementary Fig. 5a we show such averages obtained at $T = 0$ K under a normal load of 0 (black squares), 0.6 (red circles), and 1.9 (blue triangles) GPa during the first five t_0 simulations periods, each point representing an average over a t_0 time-window. It is clearly seen that steady-state is obtained already after one t_0 simulation period where the averaged friction force stabilizes. Hence, in the zero-temperature case, we discard the result of the first period and average over the rest 2-4 periods to evaluate the friction force.

For finite temperature simulations, it takes longer time for the system to reach steady state. Therefore, longer transient periods are discarded and more periods are employed to average the force traces. In this case (see Supplementary Fig. 5b, c and d), the results of each period fluctuate around an average value, and we opt to drop the first few periods (3 for 50 K and 150 K traces and 4 for the $300 - 400$ K traces) and average over the following periods ($7 - 10$ periods for the 50 K trace and $7 - 13$ periods for the $150 - 400$ K traces).

Since the friction force has a non-monotonic dependence on the normal load, we estimated the effective friction coefficients via linear fits to the force versus pressure diagrams in the low and high normal load regimes (see Supplementary Fig. S6. The data is taken from Fig. 2a in the main text.). At zero temperature, the slopes of these linear fits yield friction coefficients of 1.5×10^{-4} and -8.1×10^{-5}, respectively. Here, the friction force values obtained under normal forces of 0 and 627.2 nN were excluded from the fit, as they deviate from the linear segments and correspond to negligible friction forces. For T = 300 K, the corresponding friction coefficients are -1×10^{-5} and -8.9×10^{-5}, respectively. Overall, these values are well below the threshold of 10^{-3} for superlubric sliding.

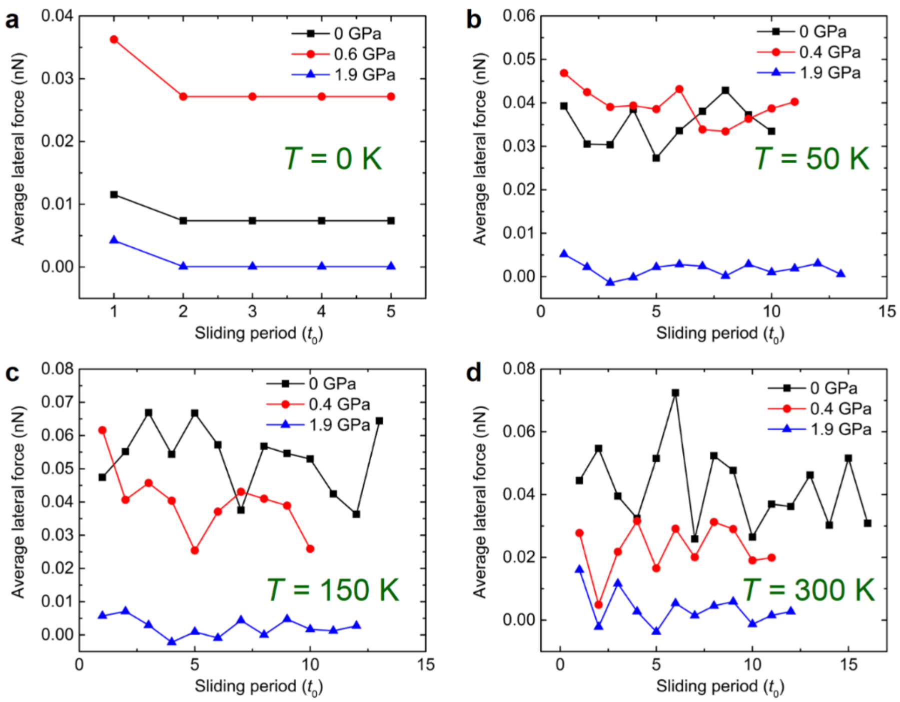

Supplementary Fig. 5. Evaluation of friction force with average lateral force for the θ_2 = 8° GB model system studied in the main text. The average lateral force as a function of sliding period for (**a**) $T = 0$ K; (**b**) 50 K; (**c**) 150 K; and (**d**) 300 K under different normal loads in the range $0 - 1.9$ GPa.

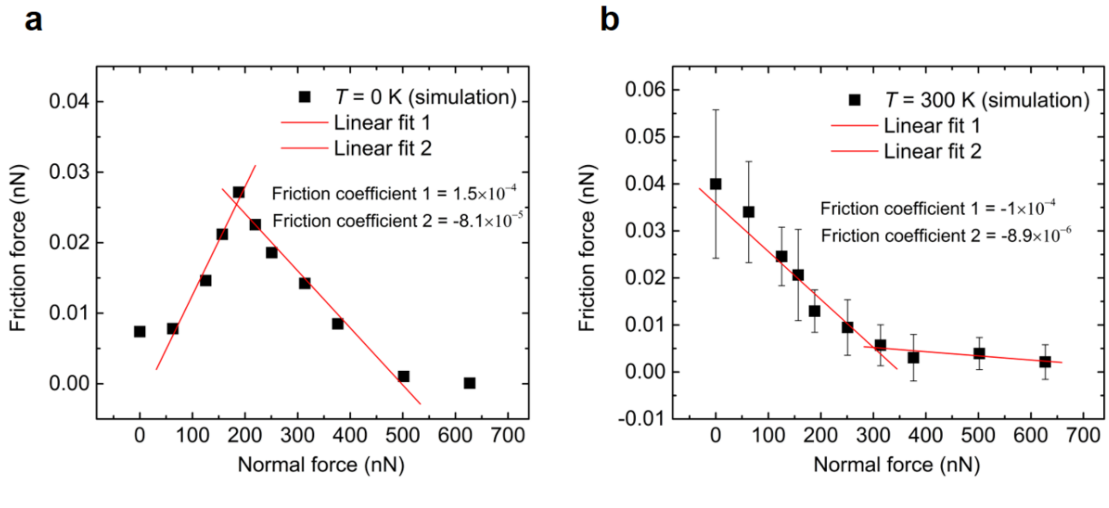

Supplementary Fig. 6. Estimation of the effective friction coefficients for θ_2 = 8° GB model system studied in the main text. Linear fits (red lines) to the low- and high-load regimes of the friction-force versus normal force curves obtained at (**a**) $T = 0$ K and (**b**) T = 300 K.

Supplementary Note 2. Frictional Dissipation Analysis

At steady state, the power invested by the slider, $p_{\text{in}} = F_f \cdot v_0$, where F_f is the friction force and v_0 is the sliding velocity, is balanced by the energy dissipation power p_{diss} through internal viscous forces. In our system, the dissipation power p_{diss} is calculated as the sum of the dissipation powers of each damped layer, written as:

$$p_{\text{diss}} = p_{\text{diss}}^{l_1} + p_{\text{diss}}^{l_2} + p_{\text{diss}}^{l_5},$$

$$p_{\text{diss}}^{l_1} = m_{\text{c}} N_{l_1} \eta \left\langle \left(v_z^{l_1}(t) \right)^2 \right\rangle,$$

$$p_{\text{diss}}^{l_2} = m_{\text{c}} \sum_i^{N_{l_2}} \sum_{j=x,y,z} \eta \left\langle \left(v_{i,j}^{l_2}(t) - v_j^{l_1}(t) \right)^2 \right\rangle, \tag{S2}$$

$$p_{\text{diss}}^{l_5} = m_{\text{c}} \sum_i^{\text{N}_{l_5}} \sum_{j=x,y,z} \eta \langle v_{i,j}^{l_5}(t)^2 \rangle,$$

where the superscript l_1, l_2, and l_5 represent the first, second, and fifth layers from the top to bottom (see Supplementary Fig. 1b) , i.e. the three damped layers, m_{c} is the atomic mass of carbon, $N_{l_{i=1,2,5}}$ is the number of atoms in the ith layer from the top, η is the damping coefficient, $v_{i,j}(t)$ is the jth Cartesian velocity component of the ith atom at time t, and $\langle \cdot \rangle$ represents steady-state time averaging. It should be noted that the first layer from top is kept rigid therefore, $v_x^{l_1}(t) = v_0$, $v_y^{l_1}(t) = 0$, and $v_z^{l_1}(t)$ are identical for all the layer atoms and dissipation occurs only in the vertical direction. Furthermore, the dissipation power of the second layer, $p_{\text{diss}}^{l_2}$, is calculated using the relative velocities of the atoms in the layer with respect to that of the rigid topmost layer.

The comparison between the molecular dynamics (MD) results of p_{in} and p_{diss} (averaged over one sliding period of ~0.6 ns), shown in Supplementary Fig. 7a, presents their general balance, further confirming that our calculations are done at the steady state. In Fig. 3a of the main text, we identify that the main channel of energy dissipation is the z direction component, which includes the contributions from all three damped layers. Here, to further elucidate the dissipation routes, we present the dissipation components in each Cartesian direction of each damped layer (see Supplementary Fig. 7b). We find that the vertical dissipative channel of the topmost layer has a

negligible contribution, while the vertical components of the second and fifth layers from top provide the major contribution to the total energy dissipation.

To understand the origin of the high out-of-plane dissipation in the second and fifth layers, we calculate the corresponding power density distributions. The calculated 2D maps of the time-averaged (over a sliding period of 0.6 ns) dissipation power density are presented in Supplementary Figs. 6-8 for 0, 0.6, and 1.9 GPa normal loads, corresponding to relatively low dissipation, peak dissipation, and very low dissipation, respectively. For zero normal load, as shown in Supplementary Fig. 8, both layers exhibit a few high dissipation power density sites (one in the second layer and two in the fifth layer from top) located above or below the GB seamline. For 0.6 GPa normal load, shown in Supplementary Fig. 9 (also shown as Fig. 3b in the main text), the number of high dissipation power density sites increases to 4 in the second layer and 6 in the fifth layer. At the higher normal of 1.9 GPa, shown in Supplementary Fig. 10, the number and power of dissipative sites is greatly reduced.

These results demonstrate that surface corrugation alone does not produce significant dissipation, and snap-through bump buckling dynamics is responsible for the enhanced friction exhibited by GBs and for the corresponding non-monotonic frictional dissipation behavior with normal load discussed in the main text.

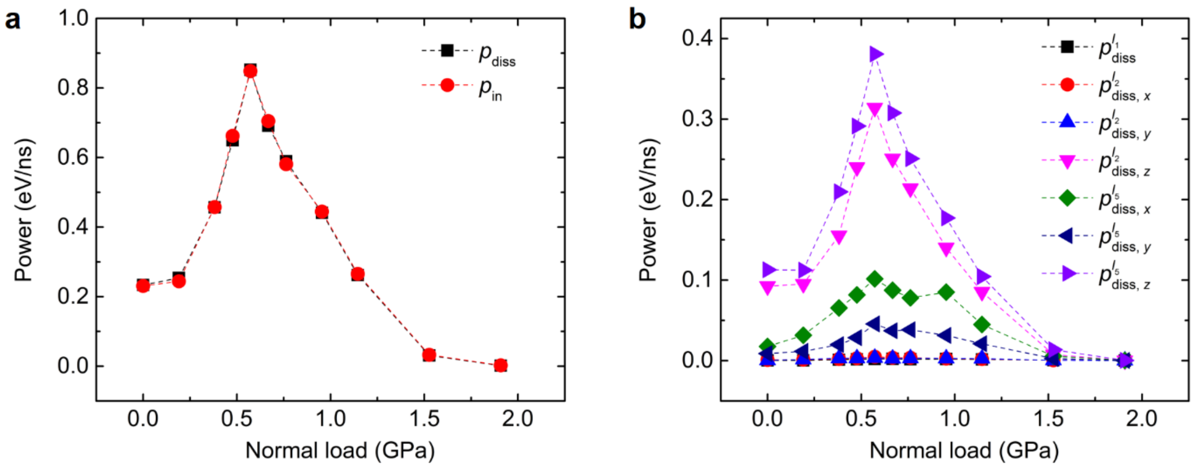

Supplementary Fig. 7. Energy dissipation via viscous damping at zero temperature. (**a**) Comparison of total input power p_{in} and dissipative power p_{diss} as a function of normal load. (**b**) The dissipation components in each Cartesian direction and each damped layer as a function of normal load.

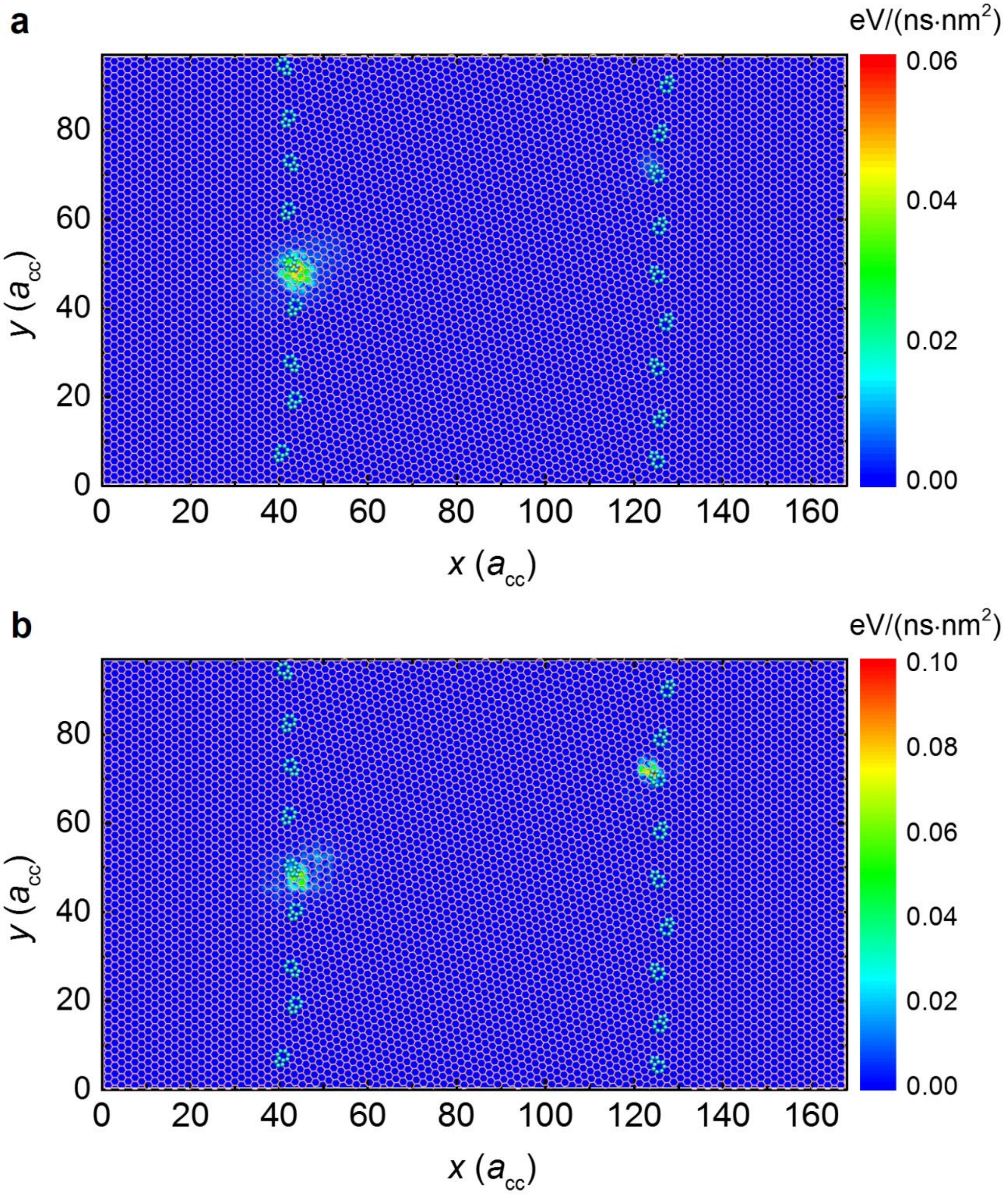

Supplementary Fig. 8. 2D maps of the time-averaged power density of the out-of-plane dissipation under zero normal load at zero temperature. (**a**) The second layer and (**b**) the fifth layer from top. The geometric configuration of the PolyGr layer is superimposed on the 2D maps. The pentagon-heptagon defects atoms are marked by cyan spheres and the hexagonal carbon atoms are represented by pink bonds. The power density map is obtained by dividing the 2D space into a grid of cells of dimensions a_{cc}^2 and summing the dissipated power of atomic sites residing within each cell.

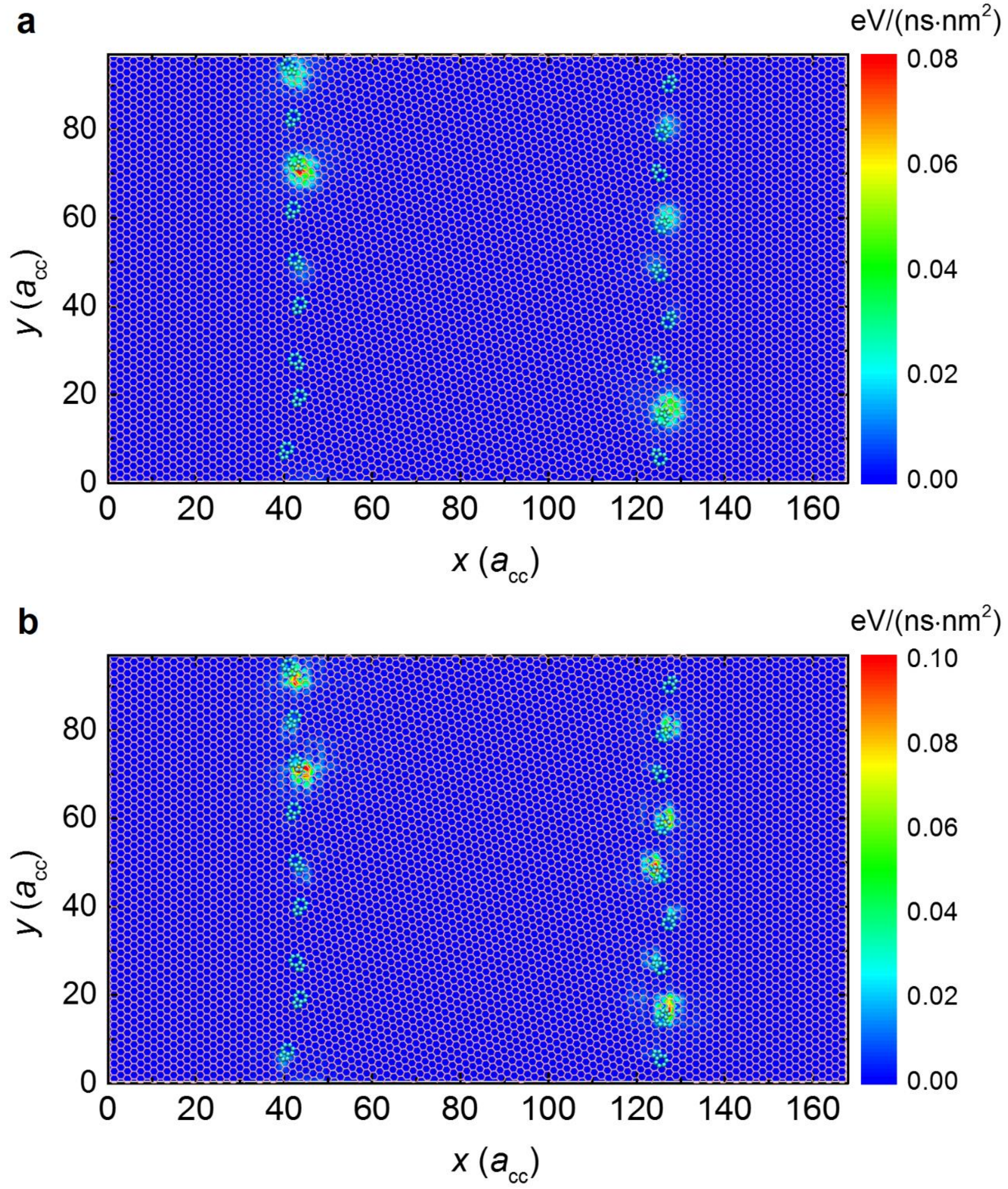

Supplementary Fig. 9. 2D maps of the time-averaged power density of the out-of-plane dissipation under a normal load of 0.6 GPa at zero temperature. (**a**) The second layer and (**b**) the fifth layer from top. The geometric configuration of the PolyGr layer is superimposed on the 2D maps. The pentagon-heptagon defects atoms are marked by cyan spheres and the hexagonal carbon atoms are represented by pink bonds. The power density map is obtained by dividing the 2D space into a grid of cells of dimensions a_{cc}^2 and summing the dissipated power of atomic sites residing within each cell.

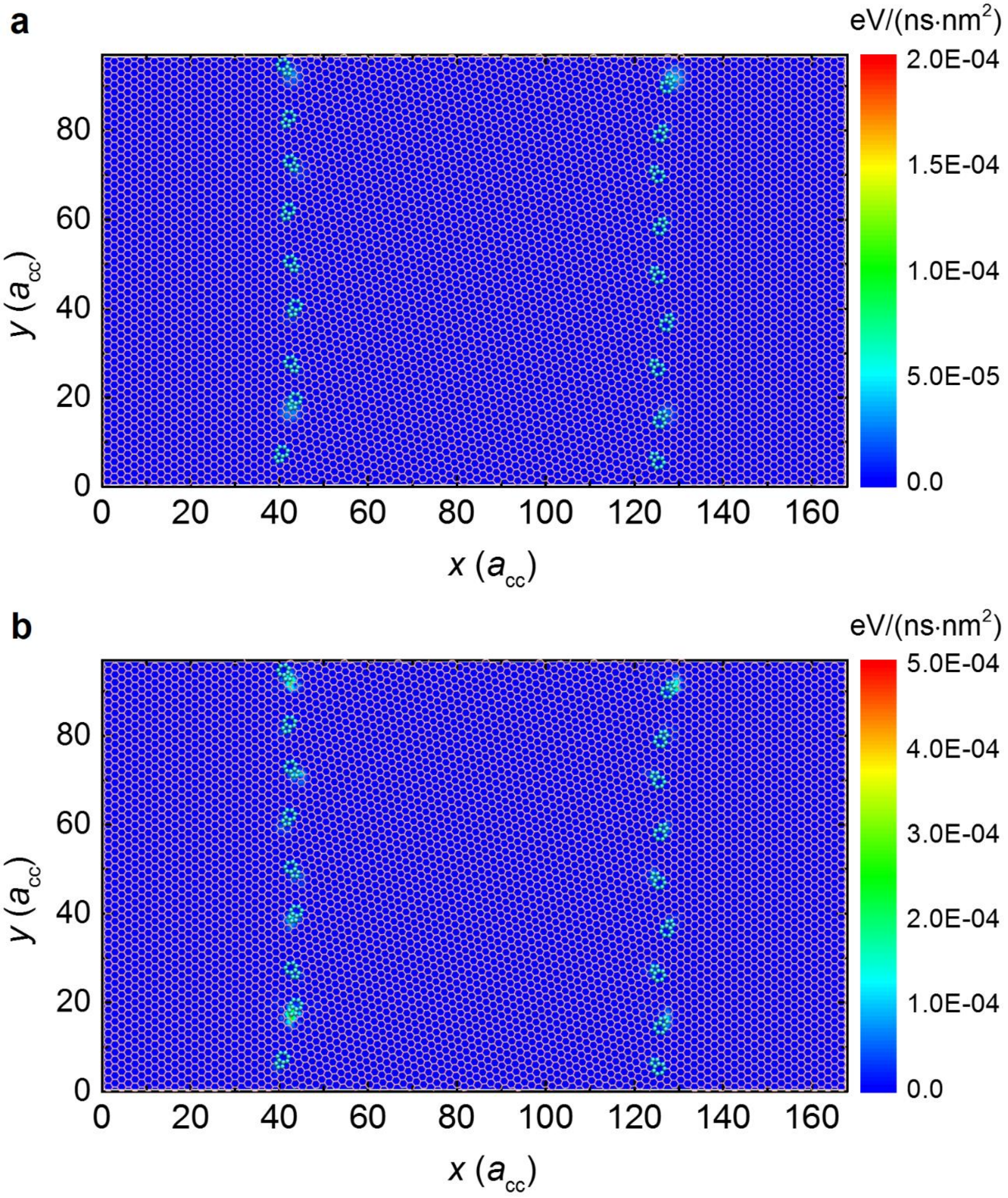

Supplementary Fig. 10. 2D maps of the time-averaged power density of the out-of-plane dissipation under a normal load of 1.9 GPa at zero temperature. (**a**) The second layer and (**b**) the fifth layer from top. The geometric configuration of the PolyGr layer is superimposed on the 2D maps. The pentagon-heptagon defects atoms are marked by cyan spheres and the hexagonal carbon atoms are represented by pink bonds. The power density map is obtained by dividing the 2D space into a grid of cells of dimensions a_{cc}^2 and summing the dissipated power of atomic sites residing within each cell.

Supplementary Note 3. Vertical Trajectories of Dislocations at Different Temperatures

Supplementary Fig. 11 presents the vertical trajectories of four representative dislocations at various temperatures under zero normal load during sliding. The trajectories show the time-dependent vertical displacement of the atom that presents the largest time-averaged root-mean-square deviation in each dislocation. The panels are ordered according to increasing buckling energy barrier from a to d. The barrier heights are calculated by spatially averaging the larger transition barrier for either upward or downward buckling (see Fig. 4a of the main text) of a given dislocation over different positions during a sliding period (similar to what was done to obtain Fig. 4b of the main text).

The trajectory presented in Supplementary Fig. 11a, corresponds to a dislocation of very small energy barrier (0.04 eV) that buckles dynamically during sliding already at zero temperature, showing well-defined periodicity of ~0.6 ns that when multiplied by the sliding velocity of $5\ \mathrm{m/s}$ yields $\sim 3\ \mathrm{nm}$ in good agreement with the periodicity of the slider in the *x* direction. When increasing the temperature to T = 50 K, the buckling frequency is enhanced due the thermal assistance. At even higher temperatures, e.g. T = 150 K and 300 K, the thermal energy becomes comparable to the energy barriers of the dislocation, leading to frequent thermally induced spontaneous buckling. For the other three dislocations with larger energy barriers (0.09, 0.15, and 0.17 eV, respectively), the sliding itself is unable to trigger buckling at zero temperature (see Supplementary Fig. 11b, c and d) and the temperature at which buckling initiates is found to grow with increasing barrier height. Once buckling occurs, its frequency (at a given temperature) is lower for dislocations exhibiting higher buckling barrier. Notably, for the two highest barrier dislocations considered (see Supplementary Fig. 11c, d) the two metastable states remain clearly evident even at a temperature of T = 400 K, indicating that the shear induced buckling mechanism remains relevant also at this temperature.

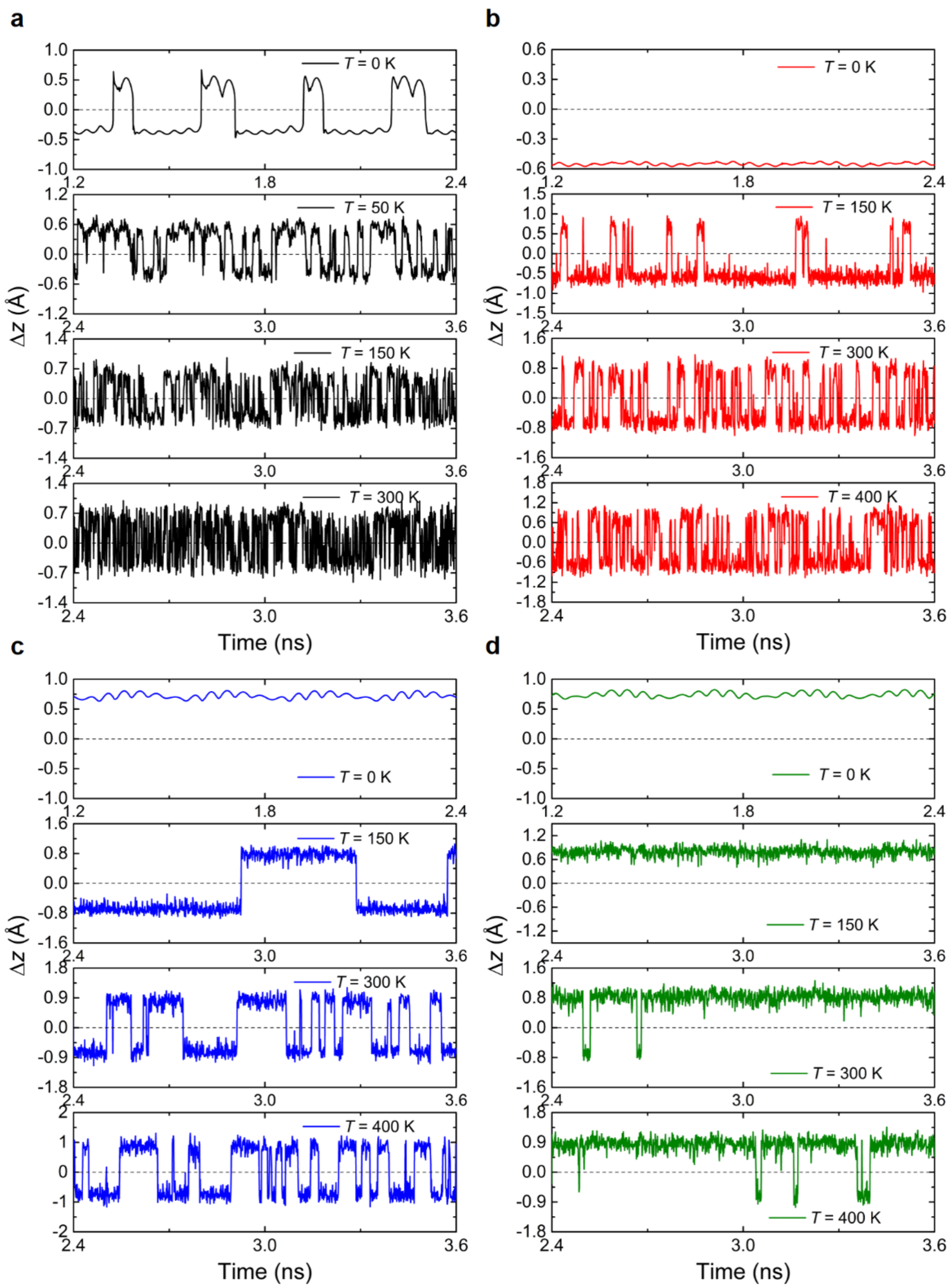

Supplementary Fig. 11. Vertical trajectories of four dislocations during sliding under zero normal load at different temperatures. The initial buckling energy barriers of the dislocations are: (**a**) 0.04, (**b**) 0.09, (**c**) 0.15, and (**d**) 0.17 eV. The trajectory of each dislocation is defined by the vertical displacement, Δz, of the atom with maximum root-mean-square corrugation with respect to the average height of the PolyGr surface, indicated by the dashed lines. The trajectories are taken from simulation results at steady state, where 1.2 ns corresponds to two sliding periods of the PrisGr slider.

Supplementary Note 4. Additional Information for Transition Energy Barriers of Dislocations

<u>Comparison of the Energy Barriers of Dislocations Obtained using Nudged Elastic Band Calculations and Equilibrium Simulations</u>

In the main text, the transition energy barriers of the two buckled states of a dislocation are extracted from the Helmholtz free energy profile $A = k_{\mathrm{B}}T \ln[\rho(\Delta z)]$, where $\rho(\Delta z)$, the probability density distribution of finding the atom with maximum root-mean-square corrugation in each dislocation at a displacement of Δz away from the average height of the two PolyGr layer grains, is calculated from the atomic trajectory during an equilibrium simulation at $T = 300$ K. These calculations were performed for fixed supercell dimensions, constant number of atoms, and constant temperature. While periodic boundary conditions are applied in all directions, a large vacuum size of 60 Å in the vertical direction was employed, effectively enforcing only lateral periodic boundary conditions. Therefore, the actual vertical dimensions of the 6-layered slab were not fixed during the calculations.

To validate the accuracy of this method, we also performed nudged elastic band (NEB) calculations[20-23] between the two buckled states of the dislocations. Since the NEB method yields reliable reaction paths at zero temperature, we use it as a benchmark for the results obtained via equilibrium simulations. To this end, following the initial annealing stage of all six modeled layers we focused on a dislocation protruding either upward or downward. This serves as one basin in the NEB calculation. Then we applied a vertical force on the atom chosen to plot the trajectory and re-optimized the geometry using the FIRE algorithm[16-17] with a force criterion of 10^{-4} eV/Å. Once the dislocation buckled, we removed the force and repeat the optimization to obtain the second NEB basin. The 14 replicas, which were connected with springs of constant 10 N/m and zero equilibrium distance, were then relaxed using the FIRE algorithm[16-17] with a force criterion of 5×10^{-5} eV/Å. The reaction coordinate was defined based on the normalized two norms of the $3N$-length vector of distances between replicas as per Ref. 24. The reaction coordinates for the first replica and the last replica were 0.0 and 1.0, corresponding to the downward and upward buckled states, respectively.

Supplementary Fig. 12 presents the comparison of the energy barriers of four chosen dislocations (in decreasing order of barrier heights) obtained from the NEB calculations and equilibrium MD

simulations. Generally, the two methods provide qualitatively similar potential energy profiles, with energy barriers that match to within ~10 meV (smaller than $k_B T$ at room temperature).

Since the equilibrium MD simulations allow us to obtain a good estimation of the buckling energy barriers despite the minor volume flexibility under normal load (with the exception of very low barriers, panels (g) and (h) of Supplementary Fig. 12) with a considerably lower computational burden compared to the NEB approach, we opted to use the former to obtain the results presented in the main text.

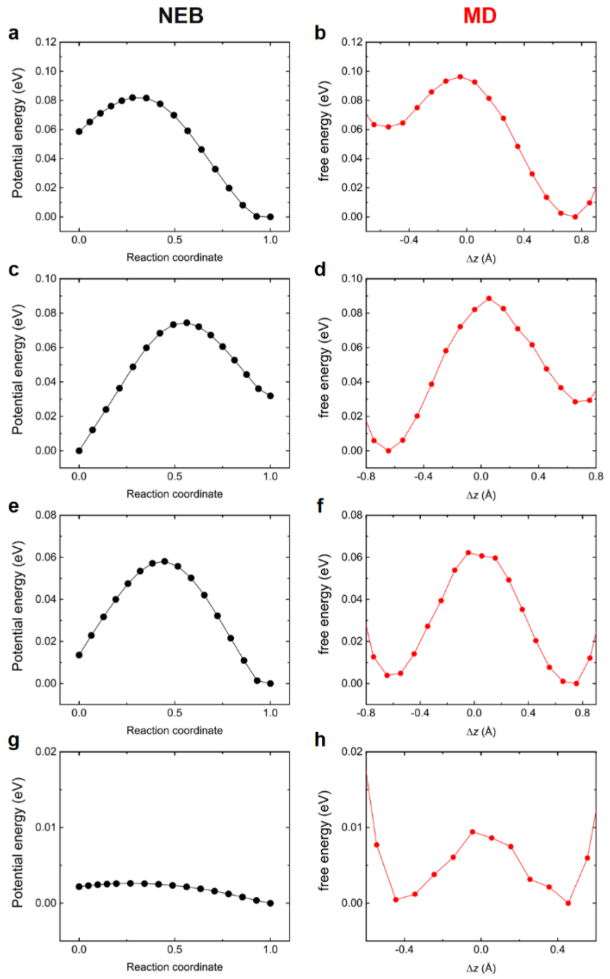

Supplementary Fig. 12. Comparison of the transition energy barriers obtained via NEB calculations and equilibrium MD simulations at *T* = 300 K under a normal load of 0.4 GPa. The left column: (**a**), (**c**), (**e**), and (**g**) NEB results of reaction paths for four chosen dislocations ordered according to decreasing barrier heights. Right column: (**b**), (**d**), (**f**) and (**h**) free energy profiles for the same dislocations obtained via equilibrium MD simulations at $T = 300$ K. The lowest energies obtained in the potential energy and free energy profiles are set to zero for comparison purposes.

Variation of the Buckling Energy Profile with the Lateral Displacement of the Slider

Supplementary Fig. 13 shows the variation of the energy profile along the buckling trajectory with the lateral displacement of the slider for four representative dislocations. These variations stem from the fact that the interlayer interactions (and hence the ILP) are registry dependent (see several representative stacking modes in Supplementary Fig. 14). At certain positions (e.g. the purple line in Supplementary Fig. 13b) the energy barrier along the buckling trajectory vanishes allowing for buckling to occur even at zero temperature. Further variations of the buckling energy profile during sliding may result in reverse buckling. This is manifested as dynamic dislocation buckling during sliding.

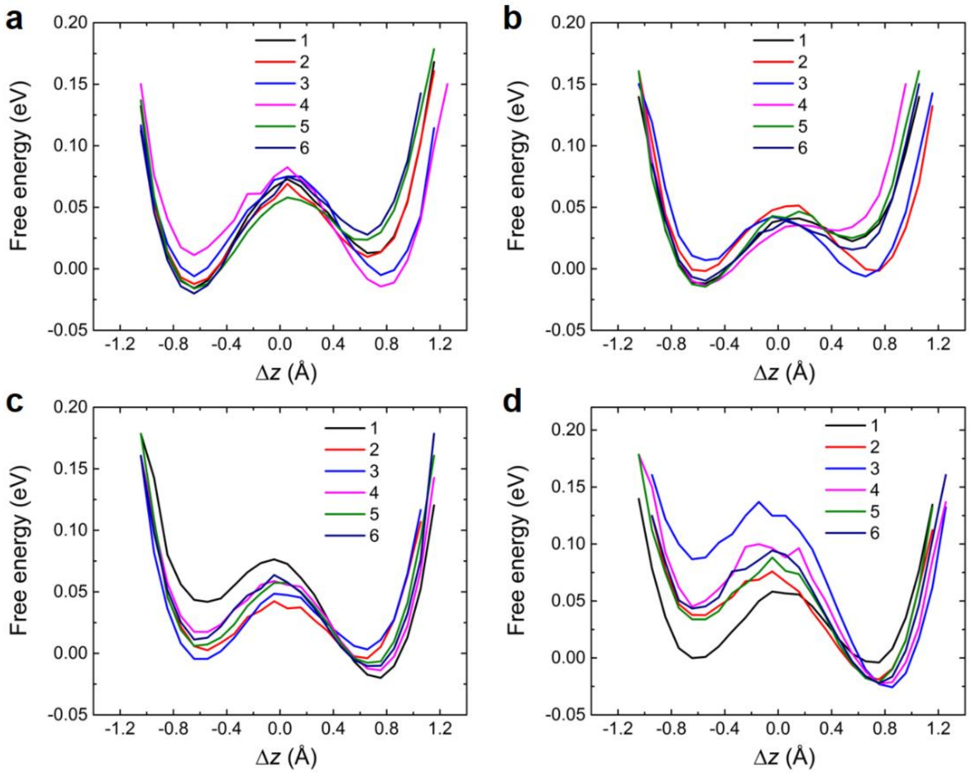

Supplementary Fig. 13. Buckling free energy profiles of four representative dislocations (a)-(d) at six equidistant slider positions denoted by the numbers 1-6 along one sliding period of 29.8 Å, under a normal load of 0.4 GPa. The buckling energy profile at position 1 in panel (a) is the same as that presented in Fig. 4(a) of the main text.

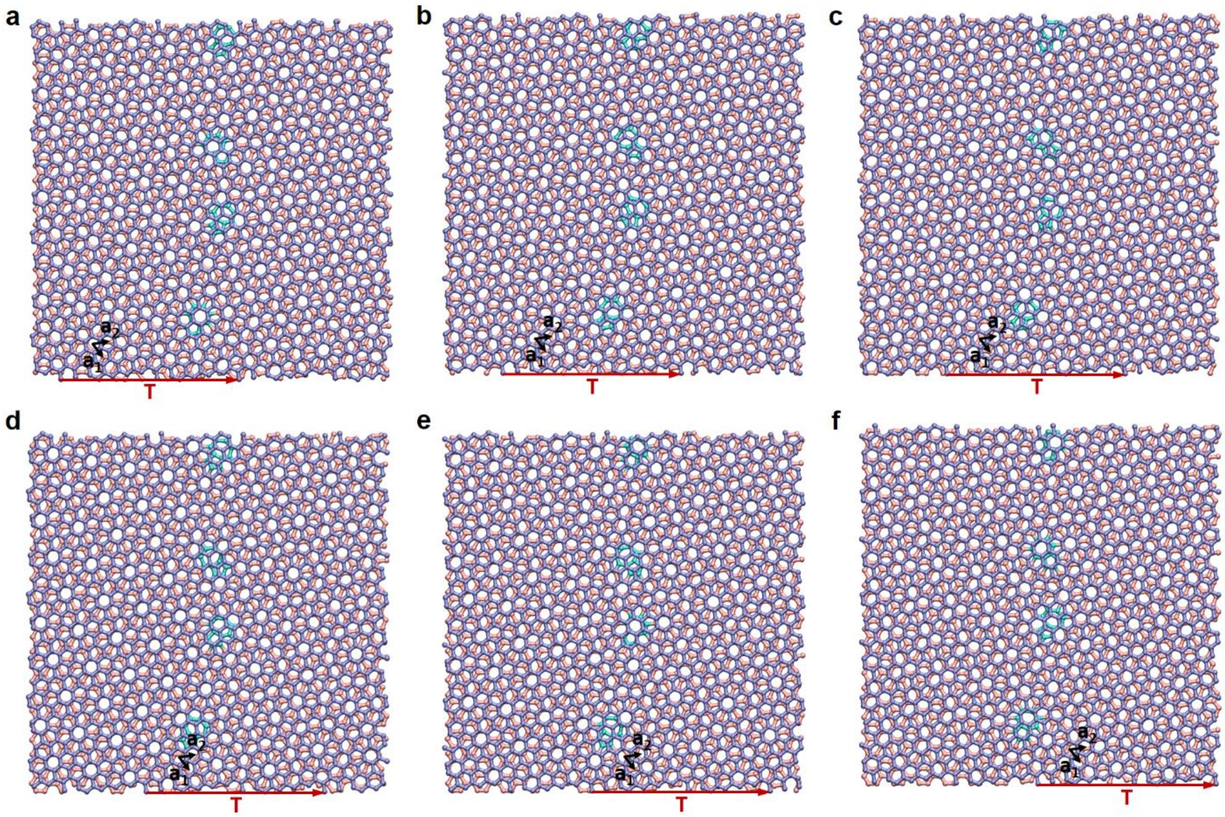

Supplementary Fig. 14. Illustration of the equidistant slider positions used for the free energy profile calculations. (a)-(f) The stacking configurations for six equidistant slider positions along one sliding period. For clarity, a zoom in on an area of $6 \times 6\ \mathrm{nm}^2$ near the bottom edge of GB 1 is presented for the top rigid slider layer (purpule bonds) and the PolyGr layer (pink and cyan bonds, representing hexagonal and dislocation atoms). The periodiciy (~ 3 nm) along the sliding direction is marked by the red arrows.

Supplementary Note 5. Effects of Stack Thickness, Sliding Velocity, and GB Misfit Angle

Effect of Stack Thickness

Considering that the stack stiffness may affect its frictional response, the frictional behavior observed in the 6-layered system should be compared to that obtained for thicker model junctions. To this end, we built two thicker stacks consisting of 8 and 10 layers (only one of which is PolyGr), half residing in the slider and half in the substrate. The geometry of each model system was first optimized and then annealed as done for the 6-layered stack. During the dynamics, damping was applied to the two layers adjacent to the outer fixed substrate layer and rigid slider layer (the second layer from the top and the second layer from the bottom of the stack). To reduce computational burden, the comparison was performed only at zero temperature. Supplementary Fig. 15(a) presents the annealed corrugated topographies of the PolyGr layer for different model system thicknesses, which are found to be very similar in all three cases considered. Minor differences in the protrusion direction may be found depending on the specific annealing realization. Supplementary Fig. 15(b) demonstrates that the load dependence of the friction is weakly sensitive to the model system thickness. Furthermore, similar dislocation buckling trajectories are obtained for different model system thicknesses (see Supplementary Fig. 15(c)-(d). This demonstrates that the 6-layered model system used to obtain the results presented in the main text can be utilized to study the frictional behavior of thicker stacks incorporating a PolyGr layer.

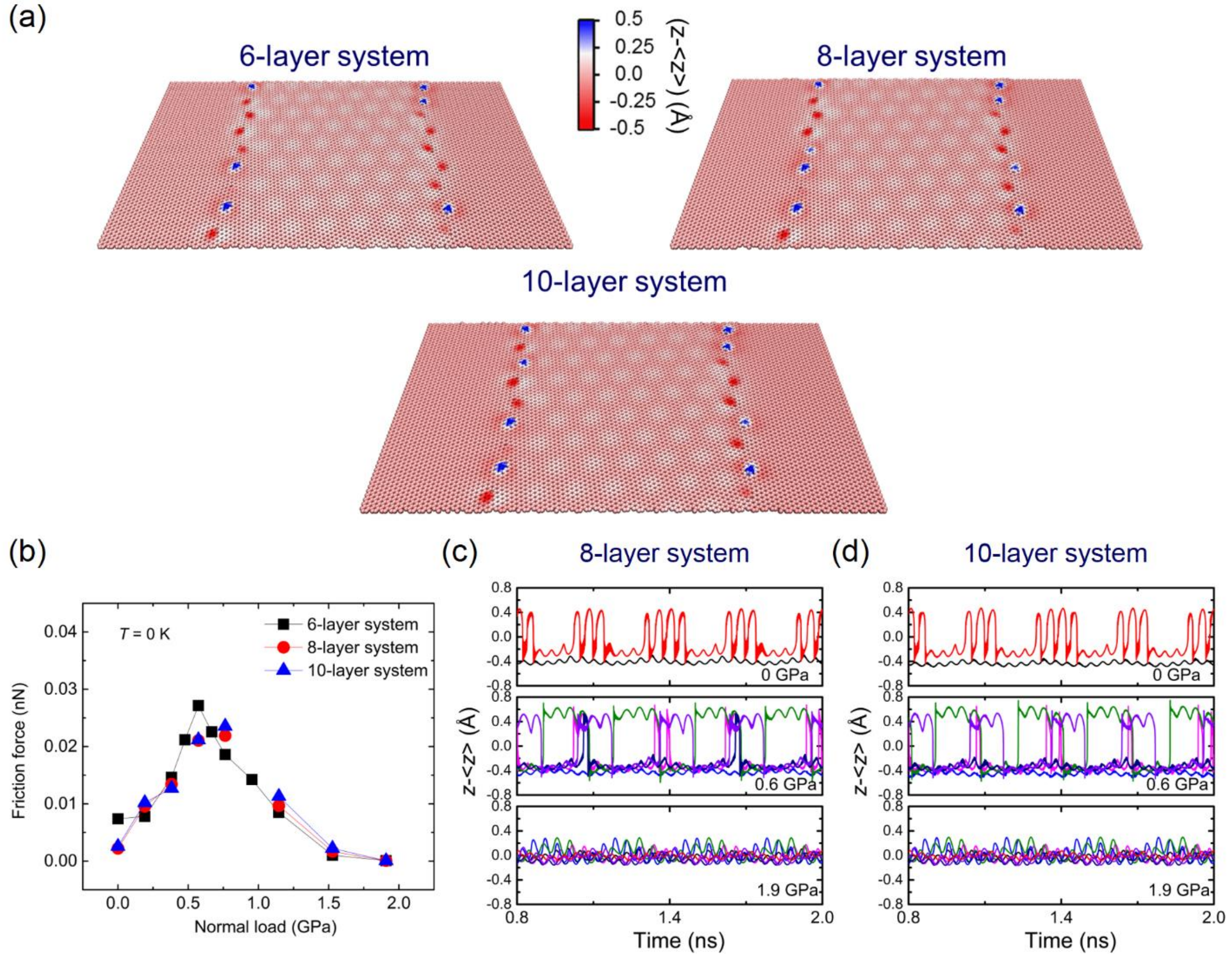

Supplementary Fig. 15. Comparison of simulation results for model systems of different thicknesses. (a) Annealed topographies of the PolyGr layer embedded within stacks of various number of layers. The color represents the atomic out-of-plane deformation (see color scale). (b) Load dependence of the friction force at zero temperature calculated for the 6- (black squares), 8- (red circles), and 10-layered (blue triangles) systems. (c)-(d) Representative dislocation trajectories taken from the (c) 8-layered system and the (d) 10-layered system, respectively, under normal loads of 0, 0.6, and 1.9 GPa (from top to bottom). The dislocations in each panel (different line colors) are chosen to be the same as those appearing in Fig. 3(c) of the main text.

Effect of Sliding Velocity

In the main text, we have presented results obtained using a slider velocity of $5\ \mathrm{m/s}$. To evaluate the sensitivity of our results and the dependence of our main conclusions on the sliding velocity, we have repeated our simulations at two additional velocities of $2\ \mathrm{m/s}$ and $10\ \mathrm{m/s}$. Similar to the $5\ \mathrm{m/s}$ case, dynamic snap-through dislocation buckling is found for both cases (see Supplementary Fig. 16), leading to a nonmonotonic to monotonic transition of the friction load dependence with increasing temperature (see panels (a)-(c) of Supplementary Fig. 17). Increasing the sliding velocity results in a mild shift of the friction force peak position toward higher normal loads. This results from the fact that at higher velocities the timescale for each individual buckling event is reduced, thus reducing the probability of thermal activation to promote buckling. In such case, a higher normal load is required for buckling to occur. Such behavior is also reproduced by our phenomenological model (see panels (d)-(f) of Supplementary Fig. 17). The fact that we find only mild quantitative (rather than qualitative) changes of the system's friction with sliding velocity suggests that the mechanism underlying the frictional behavior predicted by our simulations is insensitive to variations of the sliding velocity within the wide velocity range considered.

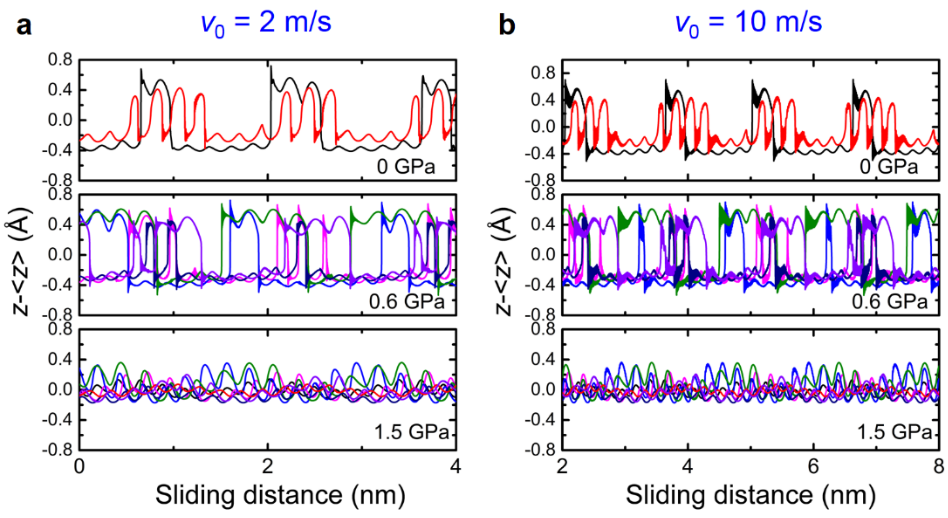

Supplementary Fig. 16. Representative GB dislocation vertical trajectories for sliding velocities of (a) $\boldsymbol{v_0} = \mathbf{2\ m/s}$ and (b) $\boldsymbol{v_0} = \mathbf{10\ m/s}$ at zero temperature and under normal loads of 0, 0.6, and 1.9 GPa (from top to bottom). The trajectories correspond to the same GB dislocations as in Fig. 3c of the main text.

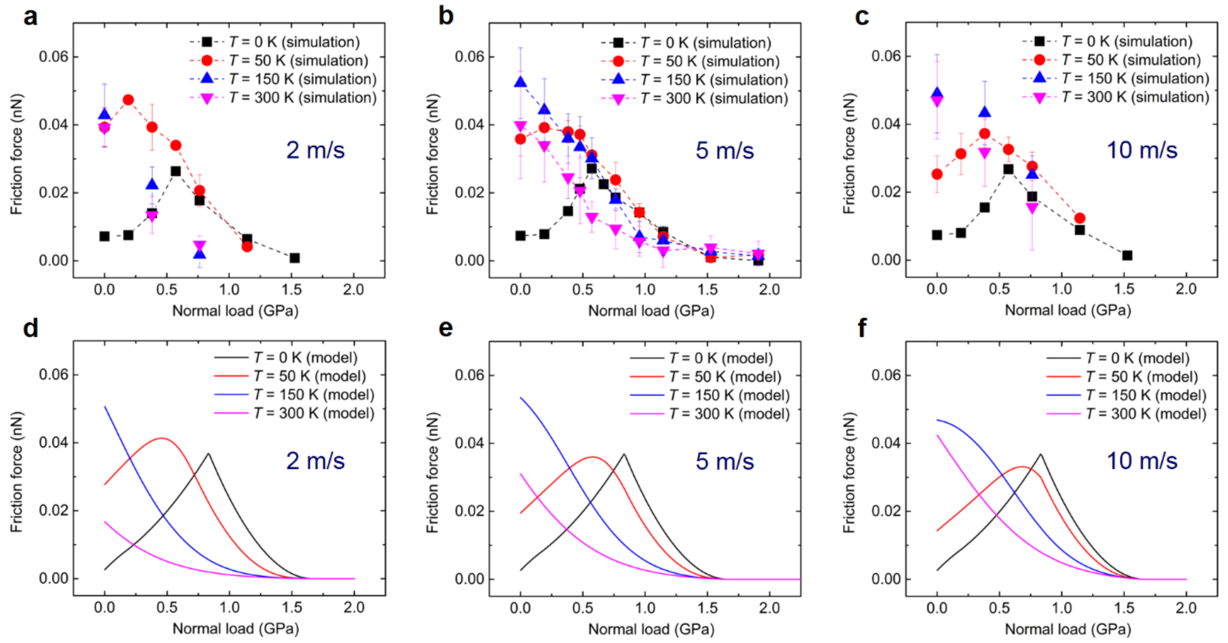

Supplementary Fig. 17. Load dependence of the friction force obtained for sliding velocities of 2, 5, and 10 $\mathrm{m/s}$. (a)-(c) MD simulation results. (d)-(f) Phenomenological model predictions. The same parameter set as in Fig. 2(c)-(d) of the main text is used. The error bars in panels a, b and c represent the standard deviations obtained from averaging friction forces over 3-13 consecutive sliding periods.

Load Dependence of the Friction Force Over a Grain Boundary of Misfit Angle $\theta_2 = 2.5°$

To consider the effect of the misfit angle of the GB on the frictional behavior, we built an additional system with periodic PolyGr of misfit angle $\theta_2 = 2.5°$. The lateral dimensions of the PolyGr are $16.62 \times 9.6\ \mathrm{nm}^2$. The top three layers, i.e. the slider, are oriented with $\theta_0 = 28°$ in their armchair direction with respect to the x axis (see Supplementary Fig. 18a). The annealing and sliding simulation protocols are the same as for the $\theta_2 = 8°$ case discussed in the main text. Supplementary Fig. 18a presents the annealed structure of the PolyGr surface with $\theta_2 = 2.5°$, which shows overall higher bump corrugation and lower dislocation density compared to the $\theta_2 = 8°$ GB system[15]. The load dependence of the friction force at different temperatures is shown in Supplementary Fig. 18b. The friction-load relation of the $\theta_2 = 2.5°$ GB system is found to be qualitatively similar to that presented by the $\theta_2 = 8°$ system, showing nonmonotonic behavior and peak shift towards lower normal loads with increasing temperature. The fact that fully monotonic decrease of friction with normal load is not obtained in the temperature range considered is

attributed to higher buckling energy barriers exhibited by this GB. For the same reason, the friction peak position of the $\theta_2 = 2.5°$ GB appears at higher normal loads than that of the $\theta_2 = 8°$ system.

Supplementary Fig. 19a presents representative vertical dislocation trajectories (see Supplementary Note 3 above for a detailed description) at $T = 0$ K and several normal loads. At 0 and 2.3 GPa normal loads, the dislocations undergo smooth variation in height during sliding, corresponding to low friction. While at 1.5 GPa normal load, the dislocations show significant dynamic buckling between the two states, which matches the maximum friction at $T = 0$ K. As the temperature is increased to 150 K and 300 K (see Supplementary Fig. 19b and c), substantial thermally induced buckling occurs already at lower normal loads of 1.0 GPa and at 0.6 GPa, respectively. This results in a friction force peak shift towards lower normal loads. Notably, at $T = 300$ K, occasional buckling due to thermal activation during sliding is also observed at 0 GPa normal load, leading to friction enhancement.

We therefore conclude that the same frictional mechanisms dictate the tribological properties of the $\theta_2 = 2.5°$ and $\theta_2 = 8°$ GB systems yielding similar frictional characteristics. This indicates the universality of the phenomenon that is expected to appear in many other corrugated GBs within polycrystalline layered materials junctions.

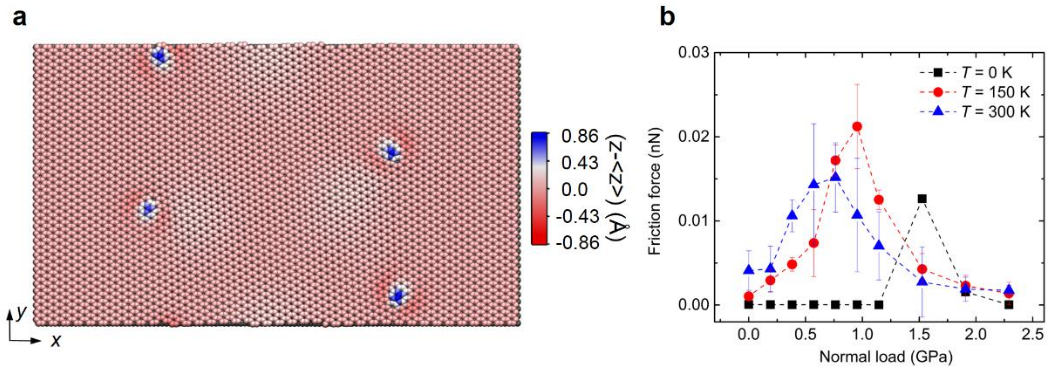

Supplementary Fig. 18. Model system and load-dependent friction force for a PolyGr junction with a misfit angle of θ_2 = 2.5°. (**a**) Top view topographic map of the annealed PolyGr layer within the six layered stack (the top three layers are not shown). False atom coloring represents the relative atomic height with respect to the average height of the grains (see color bar). (**b**) Load dependence of the friction force of this GB at various temperatures. The error bars in panel b represent the standard deviations obtained from averaging friction forces over at least 3 consecutive sliding periods.

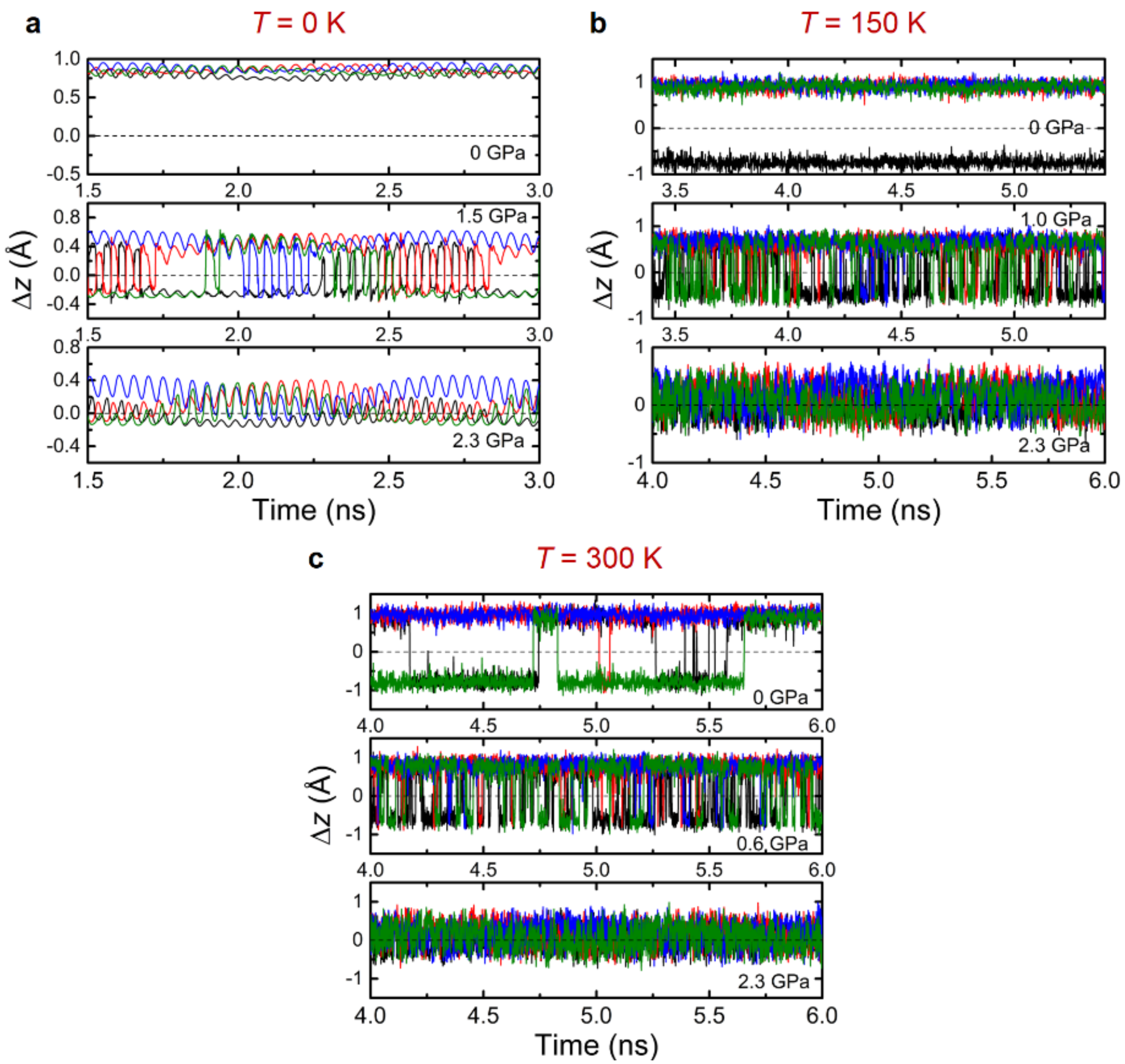

Supplementary Fig. 19. Dislocation trajectories in a PolyGr GB with misfit angle of $\theta_2 = 2.5°$ for several normal loads. The temperatures are (**a**) $T = 0$ K, (**b**) $T = 150$ K, and (**c**) $T = 300$ K. The four colored solid lines represent the four different GB dislocations (see Supplementary Fig. 11a). The dashed black line represents the average height of the two grains. For the PrisGr slider oriented at 28°, the sliding periodicity (at a velocity of 5 m/s) corresponds to ~1.1 ns.

Supplementary Note 6. Phenomenological Model

In this section, we provide the derivation of the explicit form of the dissipated energy $\Delta w(\sigma)$, within the phenomenological model, for the shear induced buckling of a given dislocation over a sliding period Δx at normal load σ and finite temperature T.

In the main text, we have shown that the energy dissipated due to the shear induced buckling of the dislocation over the sliding period is given by the following equation (Eq. (10) of the main text):

$$\Delta w(\sigma) = \int_0^{\Delta x} \mathrm{d}x [\Delta E^n_{\max}(\sigma) - \Delta E^n(x,\sigma)] f(x,\sigma) H\big(\Delta E^n(x,\sigma)\big), \tag{S3}$$

where $\Delta E^n_{\max}(\sigma) - \Delta E^n(x,\sigma)$ is the dissipated elastic energy invested in depressing the dislocation if it buckles at point x, $f(x,\sigma) = -\mathrm{d}p(x,\sigma)/\mathrm{d}x$, $p(x,\sigma)$ is given by Eq. (9) of the main text, and the Heaviside step function screens unphysical negative barrier heights. Eq. (S3) can be integrated, yielding:

$$\Delta w(\sigma) = -[\Delta E^n_{\max}(\sigma) - \Delta E^n_{\min}(\sigma)] \frac{x^*}{\Delta x} p(x^*,\sigma) +$$
$$k_B T e^{c_1 e^{-\frac{\Delta E^n_{\max}(\sigma) H\left(\Delta E^n_{\max}(\sigma)\right)}{k_B T}}} \left[E1\left(c_1 e^{-\frac{\Delta E^n_{\max}(\sigma) H\left(\Delta E^n_{\max}(\sigma)\right)}{k_B T}} \right) - E1\left(c_1 e^{-\frac{\Delta E^n_{\min}(\sigma) H\left(\Delta E^n_{\min}(\sigma)\right)}{k_B T}} \right) \right], \tag{S4}$$

where $x^* = \frac{\Delta E^n_{\max}(\sigma)\Delta x}{\Delta E^n_{\max}(\sigma) - \Delta E^n_{\min}(\sigma)\left[1 - H\left(\Delta E^n_{\min}(\sigma)\right)\right]}$ is the largest displacement for which $\Delta E^n(x,\sigma) > 0$, and $E1(x) = \int_x^\infty e^{-t} t^{-1} dt$ is the exponential integral. Next, we should consider separately two cases: (i) $x^* < \Delta x$ such that in the range $x^* < x < \Delta x$ the barrier $\Delta E^n(x,\sigma)$ vanishes, and (ii) the case where $x^* = \Delta x$ for which the barrier never vanishes during sliding. In the first case, $p(x^*,\sigma) \ll 1$ and the first term in Eq. (S4) can be neglected. In the second case, $p(x^*,\sigma) = p(\Delta x,\sigma)$. Therefore, Eq. (S4) can be approximated as:

$$\Delta w(\sigma) \approx -[\Delta E^n_{\max}(\sigma) - \Delta E^n_{\min}(\sigma)] p(\Delta x,\sigma) H\big(\Delta E^n_{\min}(\sigma)\big) +$$
$$k_B T e^{c_1 e^{-\frac{\Delta E^n_{\max}(\sigma) H\left(\Delta E^n_{\max}(\sigma)\right)}{k_B T}}} \left[E1\left(c_1 e^{-\frac{\Delta E^n_{\max}(\sigma) H\left(\Delta E^n_{\max}(\sigma)\right)}{k_B T}} \right) - E1\left(c_1 e^{-\frac{\Delta E^n_{\min}(\sigma) H\left(\Delta E^n_{\min}(\sigma)\right)}{k_B T}} \right) \right]. \tag{S5}$$

Using the explicit form of $p(\Delta x,\sigma)$ (Eq. (9) of the main text)] in Eq. (S5) we obtain:

$$\Delta w(\sigma) \approx -[\Delta E^n_{\max}(\sigma) - \Delta E^n_{\min}(\sigma)] e^{-c_1 \left\{ e^{-\frac{\Delta E^n_{\min}(\sigma) H(\Delta E^n_{\min}(\sigma))}{kT}} - e^{-\frac{\Delta E^n_{\max}(\sigma) H(\Delta E^n_{\max}(\sigma))}{kT}} \right\}} H(\Delta E^n_{\min}(\sigma)) +$$

$$k_B T e^{c_1 e^{-\frac{\Delta E^n_{\max}(\sigma) H(\Delta E^n_{\max}(\sigma))}{k_B T}}} \left[E1\left(c_1 e^{-\frac{\Delta E^n_{\max}(\sigma) H(\Delta E^n_{\max}(\sigma))}{k_B T}} \right) - E1\left(c_1 e^{-\frac{\Delta E^n_{\min}(\sigma) H(\Delta E^n_{\min}(\sigma))}{k_B T}} \right) \right], \quad \text{(S6)}$$

where we used the relation $\Delta E^n(\Delta x, \sigma) = \Delta E^n_{\min}(\sigma)$, due to the assumption of linear decrease of $\Delta E^n(x, \sigma)$ with x.

Notably, in the limit of zero temperature, using the approximation $E1(t \to 0) \approx -\gamma - \ln(t)$, where $\gamma \approx 0.5772156649$ is the Euler–Mascheroni constant, Eq. (S6) reduces to:

$$\Delta w(\sigma) \approx -[\Delta E^n_{\max}(\sigma) - \Delta E^n_{\min}(\sigma)] H(\Delta E^n_{\min}(\sigma)) + [\Delta E^n_{\max}(\sigma) H(\Delta E^n_{\max}(\sigma)) - \Delta E^n_{\min}(\sigma) H(\Delta E^n_{\min}(\sigma))] =$$

$$= -\Delta E^n_{\max}(\sigma) H(\Delta E^n_{\min}(\sigma)) + \Delta E^n_{\max}(\sigma) H(\Delta E^n_{\max}(\sigma)) = \Delta E^n_{\max}(\sigma)[H(\Delta E^n_{\max}(\sigma)) - H(\Delta E^n_{\min}(\sigma))]. \quad \text{(S7)}$$

The right-hand-side of Eq. (S7) can be further simplified by considering separately the two cases mentioned above. When $\Delta E^n_{\min}(\sigma) \le 0$ we have $\Delta w(\sigma) \approx \Delta E^n_{\max}(\sigma) H[\Delta E^n_{\max}(\sigma)]$ and when $\Delta E^n_{\min}(\sigma) > 0$ (and naturally also $\Delta E^n_{\max}(\sigma) > 0$) we have $\Delta w(\sigma) \approx -\Delta E^n_{\max}(\sigma) + \Delta E^n_{\max}(\sigma) = 0$. These two cases can be unified as follows:

$$\Delta w(\sigma) \approx \Delta E^n_{\max}(\sigma)\{1 - H[\Delta E_{\min}(\sigma)]\} H[\Delta E^n_{\max}(\sigma)], \quad \text{(S8)}$$

yielding the expression for the dissipated energy used in Eq. (2) of the main text.

Supplementary References

1 Ophus, C., Shekhawat, A., Rasool, H. & Zettl, A. Large-scale experimental and theoretical study of graphene grain boundary structures. *Phys. Rev. B* **92**, 205402, (2015).
2 Shekhawat, A. & Ritchie, R. O. Toughness and strength of nanocrystalline graphene. *Nat. Commun.* **7**, 10546, (2016).
3 Brenner, D. W. *et al.* A second-generation reactive empirical bond order (REBO) potential energy expression for hydrocarbons. *J. Phys.: Condens. Matter* **14**, 783-802, (2002).
4 Kolmogorov, A. N. & Crespi, V. H. Registry-dependent interlayer potential for graphitic systems. *Phys. Rev. B* **71**, 235415, (2005).
5 Leven, I., Azuri, I., Kronik, L. & Hod, O. Inter-layer potential for hexagonal boron nitride. *J. Chem. Phys.* **140**, 104106, (2014).
6 Leven, I., Maaravi, T., Azuri, I., Kronik, L. & Hod, O. Interlayer Potential for Graphene/h-BN Heterostructures. *J. Chem. Theory Comput.* **12**, 2896-2905, (2016).
7 Maaravi, T., Leven, I., Azuri, I., Kronik, L. & Hod, O. Interlayer Potential for Homogeneous Graphene and Hexagonal Boron Nitride Systems: Reparametrization for Many-Body Dispersion Effects. *J. Phys. Chem. C* **121**, 22826-22835, (2017).
8 Ouyang, W., Mandelli, D., Urbakh, M. & Hod, O. Nanoserpents: Graphene Nanoribbon Motion on Two-Dimensional Hexagonal Materials. *Nano Lett.* **18**, 6009-6016, (2018).
9 Krukau, A. V., Vydrov, O. A., Izmaylov, A. F. & Scuseria, G. E. Influence of the exchange screening parameter on the performance of screened hybrid functionals. *J. Chem. Phys.* **125**, 224106, (2006).
10 Tkatchenko, A., DiStasio, R. A., Car, R. & Scheffler, M. Accurate and Efficient Method for Many-Body van der Waals Interactions. *Phys. Rev. Lett.* **108**, 236402, (2012).
11 Ouyang, W. *et al.* Mechanical and Tribological Properties of Layered Materials under High Pressure: Assessing the Importance of Many-Body Dispersion Effects. *J Chem Theory Comput* **16**, 666-676, (2020).
12 Yazyev, O. V. & Louie, S. G. Topological defects in graphene: Dislocations and grain boundaries. *Phys. Rev. B* **81**, 195420, (2010).
13 Červenka, J. & Flipse, C. F. J. Structural and electronic properties of grain boundaries in graphite: Planes of periodically distributed point defects. *Phys. Rev. B* **79**, 195429, (2009).
14 Tison, Y. *et al.* Grain Boundaries in Graphene on SiC($000\bar{1}$) Substrate. *Nano Lett.* **14**, 6382-6386, (2014).
15 Gao, X., Ouyang, W., Hod, O. & Urbakh, M. Mechanisms of frictional energy dissipation at graphene grain boundaries. *Phys. Rev. B* **103**, 045418, (2021).
16 Bitzek, E., Koskinen, P., Gähler, F., Moseler, M. & Gumbsch, P. Structural Relaxation Made Simple. *Phys. Rev. Lett.* **97**, 170201, (2006).
17 Guénolé, J. *et al.* Assessment and optimization of the fast inertial relaxation engine (fire) for energy minimization in atomistic simulations and its implementation in lammps. *Comput. Mater. Sci.* **175**, 109584, (2020).
18 Ouyang, W., Ma, M., Zheng, Q. & Urbakh, M. Frictional Properties of Nanojunctions Including Atomically Thin Sheets. *Nano Lett.* **16**, 1878-1883, (2016).
19 Plimpton, S. Fast Parallel Algorithms for Short-Range Molecular Dynamics. *J. Comput. Phys.* **117**, 1-19, (1995).
20 Henkelman, G. & Jónsson, H. Improved tangent estimate in the nudged elastic band method for finding minimum energy paths and saddle points. *J. Chem. Phys.* **113**, 9978-9985, (2000).
21 Henkelman, G., Uberuaga, B. P. & Jónsson, H. A climbing image nudged elastic band method

for finding saddle points and minimum energy paths. *J. Chem. Phys.* **113**, 9901-9904, (2000).
22 Nakano, A. A space–time-ensemble parallel nudged elastic band algorithm for molecular kinetics simulation. *Comput. Phys. Commun.* **178**, 280-289, (2008).
23 Maras, E., Trushin, O., Stukowski, A., Ala-Nissila, T. & Jónsson, H. Global transition path search for dislocation formation in Ge on Si(001). *Comput. Phys. Commun.* **205**, 13-21, (2016).
24 *neb command — LAMMPS documentation*, https://lammps.sandia.gov/doc/neb.html.
