# Supplementary material for: Superlubric polycrystalline graphene interfaces
Source: Nat Commun. 2021 Sep 28;12:5694. doi: 10.1038/s41467-021-25750-w (PMC8479137; doi:10.1038/s41467-021-25750-w)
Supplement: Supplementary file 3 — Description of Additional Supplementary Files [file 41467_2021_25750_MOESM3_ESM.pdf]

Description of Additional Supplementary Files

File Name: Supplementary Movie 1
Description: 1 ns sliding simulation at zero normal load and zero temperature. The movie first shows the dynamics of all six layers for 200 ps, where the shear plane is clearly seen. To clearly demonstrate the buckling process, the simulation is then repeated for 1 ns while removing the slider layers and coloring the PolyGr layer atoms according to their out-of-plane position with respect to the average height of the two grains. Under these conditions, two dislocations undergo dynamic snap-through buckling during sliding.

File Name: Supplementary Movie 2
Description: 1 ns sliding simulation at 0.6 GPa normal load and zero temperature. When increasing the normal load to 0.6 GPa, more dislocations (~ 8) undergo dynamic snap-through buckling during sliding. At zero temperature the friction force reaches its maximum at this normal load.

File Name: Supplementary Movie 3
Description: 1 ns sliding simulation at 1.9 GPa normal load and zero temperature. With further increase of the normal load to 1.9 GPa, the height of the dislocations varies smoothly during sliding, and no snap-through buckling is observed. For this normal load the friction force is much lower compared to the 0 and 0.6 GPa cases.

File Name: Supplementary Movie 4
Description: 1 ns sliding simulation at 0.2 GPa normal load and a temperature of 50 K. These conditions correspond to the maximum value of the friction force at 50 K. Dislocations that cannot buckle at zero temperature exhibit buckling with the assistance of thermal fluctuations.

File Name: Supplementary Movie 5
Description: 1 ns sliding simulation at 0 GPa normal load and a temperature of 150 K. At 150 K, most dislocations can buckle already under zero normal load, demonstrating a significant effect of thermal activation on the shear induced buckling.

File Name: Supplementary Movie 6
Description: 1 ns sliding simulation at 0 GPa normal load and a temperature of 300 K. At 300 K, all dislocations buckle even under zero normal load during sliding. Under these conditions some dislocations exhibit high frequency buckling between upward and downward protrusion states, which is dominated by thermal fluctuations, and can be referred to as spontaneous buckling. Correspondingly, less energy is invested by the sheared slider to induce dislocation buckling, and the friction force reduces compared to that obtained at a temperature of 150 K.
